# Salt-inducible expression of OsJAZ8 improves resilience against salt-stress

**DOI:** 10.1186/s12870-018-1521-0

**Published:** 2018-11-29

**Authors:** Preshobha K. Peethambaran, René Glenz, Sabrina Höninger, S. M. Shahinul Islam, Sabine Hummel, Klaus Harter, Üner Kolukisaoglu, Donaldo Meynard, Emmanuel Guiderdoni, Peter Nick, Michael Riemann

**Affiliations:** 1 Karlsruhe Institute of Technology, Botanical Institute, Karlsruhe, Germany; 2 University Tübingen, Zentrum für Molekularbiologie der Pflanzen (ZMBP), Plant Physiology, Tübingen, Germany; 3 Centre de coopération internationale en recherche agronomique pour le développement (CIRAD), unité mixte de recherche (UMR) Amélioration Génétique et Adaptation des Plantes méditerranéennes et tropicales (AGAP), 34398 Montpellier, France; 4 Univ Montpellier, Cirad, Inra, Montpellier SupAgro, Montpellier, France

**Keywords:** BY-2 cells, Jasmonate, MeJA, OsJAZ8, Rice, Salinity, ZOS3–11, Auxin

## Abstract

**Background:**

Productivity of important crop rice is greatly affected by salinity. The plant hormone jasmonate plays a vital role in salt stress adaptation, but also evokes detrimental side effects if not timely shut down again. As novel strategy to avoid such side effects, OsJAZ8, a negative regulator of jasmonate signalling, is expressed under control of the salt-inducible promoter of the transcription factor ZOS3–11, to obtain a transient jasmonate signature in response to salt stress. To modulate the time course of jasmonate signalling, either a full-length or a dominant negative C-terminally truncated version of OsJAZ8 driven by the ZOS3–11 promoter were expressed in a stable manner either in tobacco BY-2 cells, or in *japonica* rice.

**Results:**

The transgenic tobacco cells showed reduced mortality and efficient cycling under salt stress adaptation. This was accompanied by reduced sensitivity to Methyl jasmonate and increased responsiveness to auxin. In the case of transgenic rice, the steady-state levels of *OsJAZ8* transcripts were more efficiently induced under salt stress compared to the wild type, this induction was more pronounced in the dominant-negative OsJAZ8 variant.

**Conclusions:**

The result concluded that, more efficient activation of OsJAZ8 was accompanied by improved salt tolerance of the transgenic seedlings and demonstrates the impact of temporal signatures of jasmonate signalling for stress tolerance.

**Electronic supplementary material:**

The online version of this article (10.1186/s12870-018-1521-0) contains supplementary material, which is available to authorized users.

## Background

Salinity has become one of the major abiotic stresses limiting the production of rice worldwide and, thus, has an exceptional agricultural impact: More than 280 million hectares of land are affected by salinity, and this number increases by approximately 2 million hectares, because arable land becomes uncultivable due to excess salinity each year, which overall means a global yield loss of 45–70% [1]. In order to combat salinity-dependent damage by breeding or biotechnological strategies, it is essential to understand, how plants adapt to salt stress by selective exclusion of ions, accumulation of ions into vacuoles, synthesis of osmoprotectants, induction of antioxidative enzymes, and adaptive regulation of plant hormones [2].

The phytohormone jasmonic acid (JA) has been found to increase under salt stress in rice roots, and exogenous JAs were reported to modulate salinity-induced changes of gene expression [3]. Exogenous JAs improved salt-stress tolerance in rice [4] and soybean [5]. Therefore, JA signalling is thought to play a vital role in the adaptation to salt stress but also other types of abiotic stress factors [6–9]. This notion is supported by the observation that JA biosynthesis rice mutants *(cpm2* and *hebiba*) impaired in the function of enzyme ALLENE OXIDE CYCLASE (AOC) show improved tolerance to salt stress [10], but also to drought stress [7]. Conversely, rice plants overexpressing *CYP94C2b* (encoding a JA-catabolising enzyme) show decreased JA content along with improved performance on high concentrations of salt [11]. Similarly it has been shown that constitutive overexpression of JAZ genes leads to improved abiotic stress tolerance [12].

JA signalling requires the biologically active conjugate of JA with the amino acid isoleucine (Ile) which is synthesized from the inactive JA catalysed by JAR1 (jasmonate resistant 1), a JA-amido synthetase [13]. In the absence of JA-Ile, JAZ proteins which form homo- or heterodimers, repress the transcriptional activity and turn off the expression of the early JA-responsive genes by binding to bHLH transcription factors (e.g. MYC2, MYC3, MYC4 and MYC5) that are activators of JA responses. In response to elevated JA levels due to stimulation by various stress factors, JAZ proteins are degraded in an SCF (for SKP1-CUL1-F-box)-type ubiquitin ligase SCF^COI1^-dependent manner via the 26S proteasome, leading to the rapid activation of JA responses, such as the expression of JA-responsive genes [14–16] and subsequently, the hormone signalling is attenuated by feedback reaction by induction of the JA-responsive *JAZ* genes to avoid the negative effect of over-activation of JA responses [17–19]. It is known now that the highly conserved C-terminal Jas domain of the JAZ protein mediates JAZ degradation and plays a key role in destabilizing JAZ repressors as several reports have shown that C-terminal truncated JAZ proteins (JAZΔC) are more stable in the presence of JA and shows JA-insensitive phenotype [14, 16]. This dominant action of JAZΔC may be because the protein interacts and represses the activity of MYC2 but fails to interact with COI1 [20] but this point is still unclear and yet to be confirmed.

The rapid response of jasmonate signalling conveyed by the rapid proteolytic degradation of JAZ repressors must, at one point, lead to transcriptional reprogramming culminating in the expression of adaptive or protective proteins. There is evidence that transcription factors of the Cys2His2-type zinc finger transcription factors also known as classical or TFIIIA-type finger are relevant here: Specific members of the *Arabidopsis* Cys2His2-type zinc finger family were upregulated by abiotic stress factors like drought, or salt, as well as by ABA, and overexpression of STZ improved resistance to heat, drought and salinity [21]. Likewise, *Arabidopsis* STZ was able to functionally complement salt-sensitive calcineurin mutants of yeast [22]. Also, the rice homologues of these genes were shown to confer improved tolerance to abiotic stress upon overexpression [23–26]. Thus, the salt-inducible expression of this class of transcription factors seems to be a pivotal event in the adaptation to salt stress.

Jasmonic acid regulates numerous and quite diverse plant responses leading to the question, how specificity is ensured. This aspect holds true for other generic stress signals as well leading to the concept that differences in the temporal patterns of activation are responsible for the required specificity. Such signature models have been proposed for calcium (reviewed in [27], or for oxidative stress signalling (reviewed in [28]. Also, for jasmonate signalling, such a signature model has been elaborated, reviewed in [29]. The transcriptional activation of *JAZ* genes as negative regulators of JA signalling is among the earliest responses to JA-Ile. Thus, efficient and timely activation of JA signalling will lead to a transient JA signature, activating cellular adaptation to salt stress, for instance, by activation of vacuolar sodium sequestration. In contrast, constitutive presence of JA activates programmed cell death. So, it is solely not the presence or absence of JAs alone that decides the response to salinity as adaptive or destructive, but temporal signature, amplitude of the JA signalling and cross-talk with other signalling pathways. However, so far, the evidence for such a jasmonate signature has remained correlative.

To shift to a deeper level of analysing, it would be necessary to modulate temporal patterns of jasmonate signalling rather than to constitutively disactivate (jasmonate deficient mutants) or overactivate (treatment with exogenous jasmonate, overexpression of jasmonate synthesis or signalling genes). In our current study, we designed a strategy to shift the temporal signature of JA signalling specifically under salt stress, avoiding the disadvantages of a general loss of JA signalling, such as impaired fertility [30]. As tool, we use OsJAZ8 and its dominant-negative variant OsJAZ8ΔC (where the Jas domain has been deleted). Overexpression of this truncated version exhibited a JA-insensitive phenotype [31]. To achieve expression of the full-length and the dominant negative C-terminally truncated (Jas domain lacking) JAZ8 protein under salt stress, we placed the two constructs under the control of the promoter of the CysHis2 Zinc finger transcription factor ZOS3–11. Rice ZOS3–11 and ZOS3–12 show close homology with STZ/ZAT10 and are highly induced under salt stress. As a proof of principle of our hypothesis, we first investigated our concept by stable expression in tobacco BY-2 cells as heterologous system. This allowed us to study the cellular aspects of salt stress including cell-cycle and cell expansion. We were able to monitor the activity of the promoter and to see that induction of *OsJAZ8* gene leads to a jasmonate-insensitive phenotype accompanied by better performance of cell division and viability under salt stress. Surprisingly, the jasmonate insensitivity was accompanied by amplified auxin responsiveness. In a second step, we expressed these constructs in rice itself. These transgenic rice lines show better performance under salt stress linked with high basal levels of the OsJAZ8 transcripts and high expression under salt stress. These findings provide a proof of concept for improved performance under salt stress in consequence of a modulated signature of JA signalling.

## Results

### Sequence analysis of ZOS3–11 and ZOS3–12

The rice genome contains codes for 189 zinc finger proteins (ZFPs) and among them, 179 genes have been studied [32, 33]. Rice Cys2His2 type transcription factors were selected based on various studies reported which were upregulated in salt stress. To know which ZFPs close homology with STZ/ZAT10 of Arabidopsis, phylogenetic tree was constructed using Neighbour-Joining method with the full-length amino acid sequence of all the selected proteins. Phylogenetic analysis (Additional file 1) revealed that ZOS3–11 and ZOS3–12 are homologous to STZ in *Arabidopsis*.

### Jasmonate dependent ZOS3–11 and ZOS3–12 localizes in nucleus and binds to A(G/C)T sequence

**Fig. 1:**
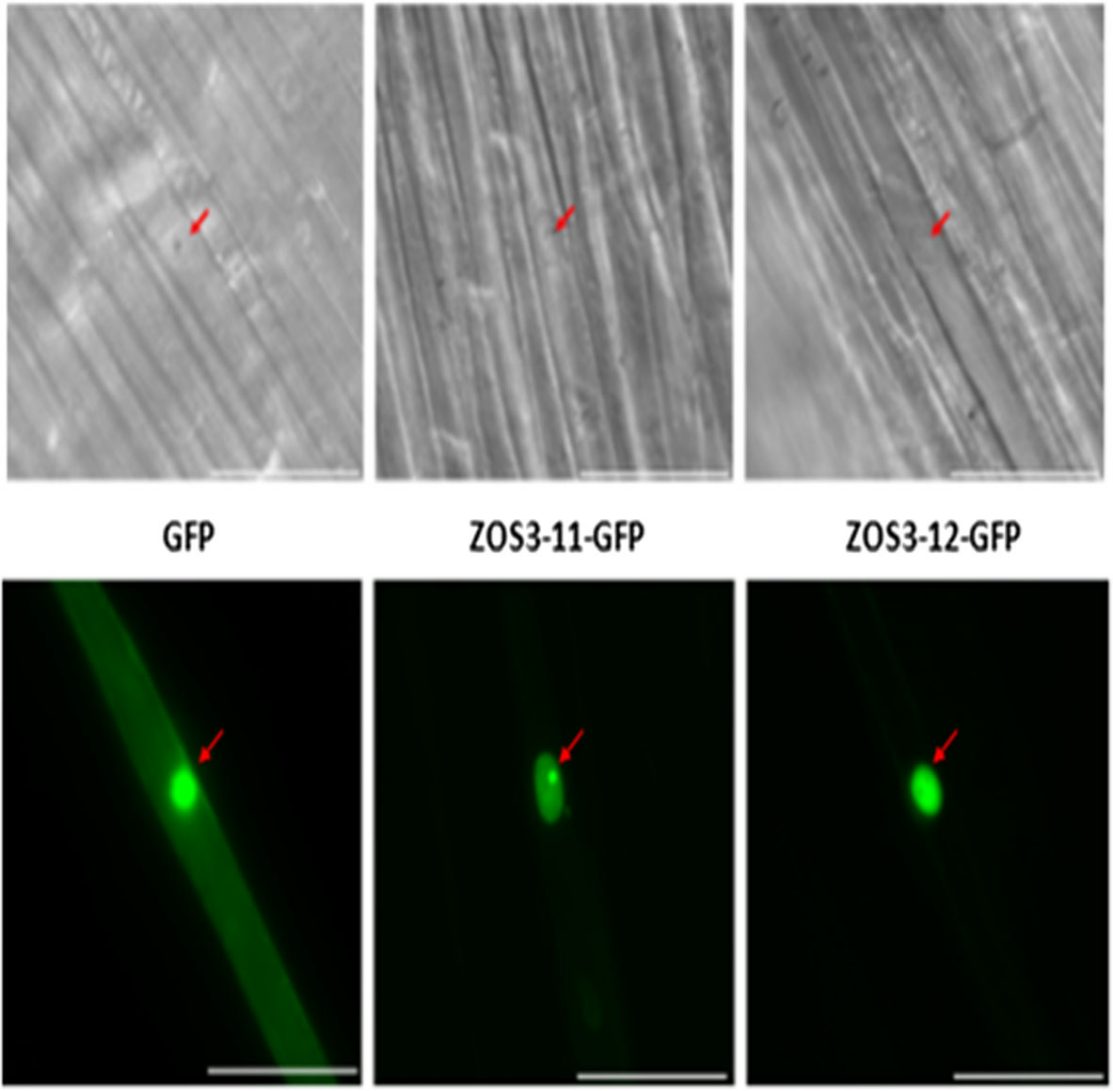
Localization of ZOS3–11 and ZOS3–12 in cells of rice coleoptile. GFP (left) and the fusion constructs ZOS3–11-GFP (centre) and ZOS3–12-GFP (right) were transiently expressed under the control of the CaMV 35S promoter. Shown are DIC (top) and fluorescence images (bottom) of each transformed cell 12–20 h after the biolistic transformation (scale bar represents 50 μm). The position of the nucleus is shown by an arrow. Three biological replicates were tested for each line

Salt tolerance zinc finger transcription factor (STZ) of *Arabidopsis* has an NLS (nuclear localization sequence) and is localized in the nucleus [34]. In the multiple sequence analysis, the two proteins ZOS3–11 and ZOS3–12 lacked NLS (Additional file 1). To examine the subcellular localization of these proteins, ZOS3–11/12-GFP fusion proteins were transiently expressed under the control of the cauliflower mosaic virus (CaMV) 35S promoter in rice coleoptile (Fig. 1) and BY-2 (Additional file 1) cells and confirmed that the fusion proteins specifically localizes to the nucleus even though they lacked a cannonical NLS domain.

**Fig. 2:**
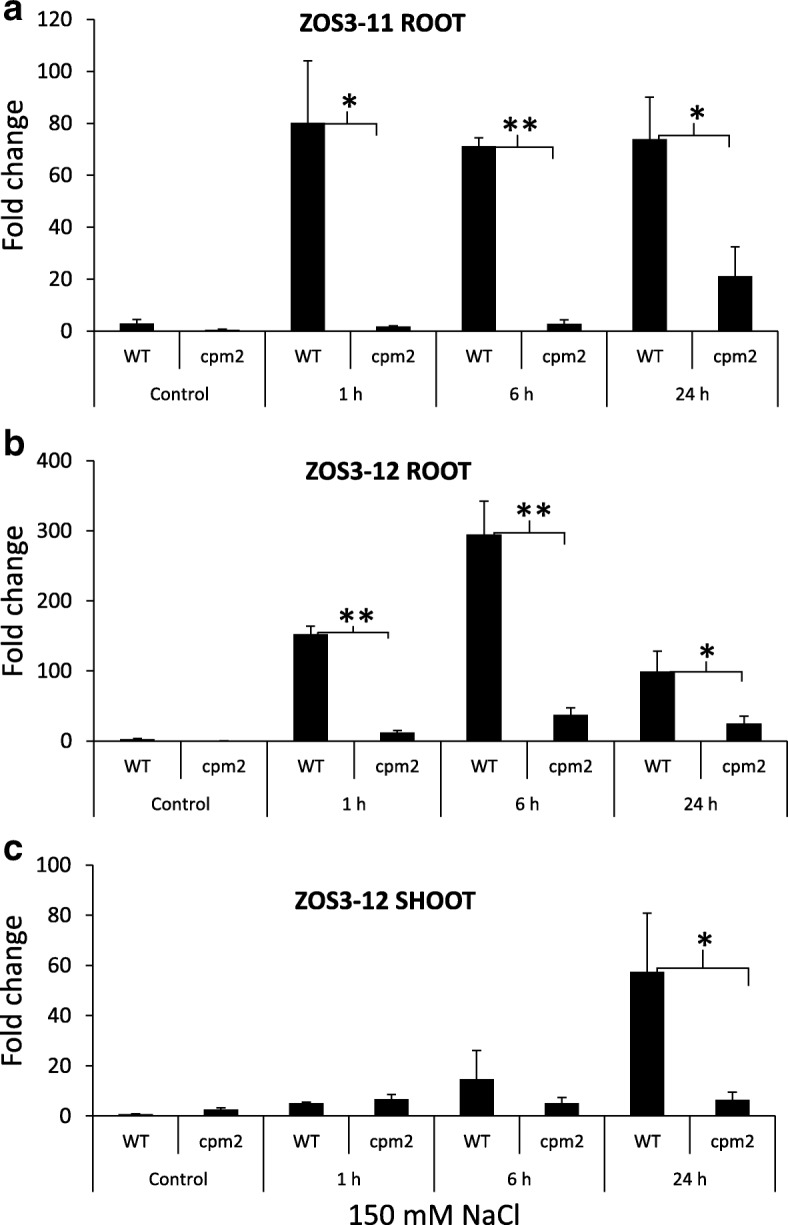
Relative gene expression analysis of (**a**) *ZOS3–11* in root and (**b**) *ZOS3–12* in root and (**c**) *ZOS3–12* in shoot under salt stress. Rice wild type (Nihonmasari) and jasmonate mutant *cpm2* were treated with 150 mM NaCl solution or water (Control). The samples were collected (1 h, 6 h and 24 h) after treatment. The relative amount of transcript was determined by normalization of the housekeeping genes EF-1α and UBQ5. Data represent average of three biological replicates with three technical replicates in each experiment. Error bars show the standard error value. The asterisk shows a significant difference between WT and *cpm2* (*, *P* < 0.05; **, *P* < 0.01) by Student’s t-test

The effect of salt stress on the expression of *ZOS3–11* and *ZOS3–12* was investigated in root and leaves using real-time PCR. The analysis revealed significantly high expression of *ZOS3–11* and *ZOS3–12* in roots of 150 mM salt treatment in WT and the expression resumed continuously even at 24 h after the salt treatment in case of *ZOS3–11* (Fig. 2a) but the expression for *ZOS3–12* decreased considerably after 6 h (Fig. 2b). On the other hand, *ZOS3–12* was expressed comparatively less in the leaves at 24 h after treatment (Fig. 2c) and there was no detectable amount of *ZOS3–11* in leaves*.* The expression level of both transcripts was considerably lower in jasmonate mutant *cpm2*, suggesting that the expression level of these two genes is dependent on jasmonate.

**Fig. 3:**
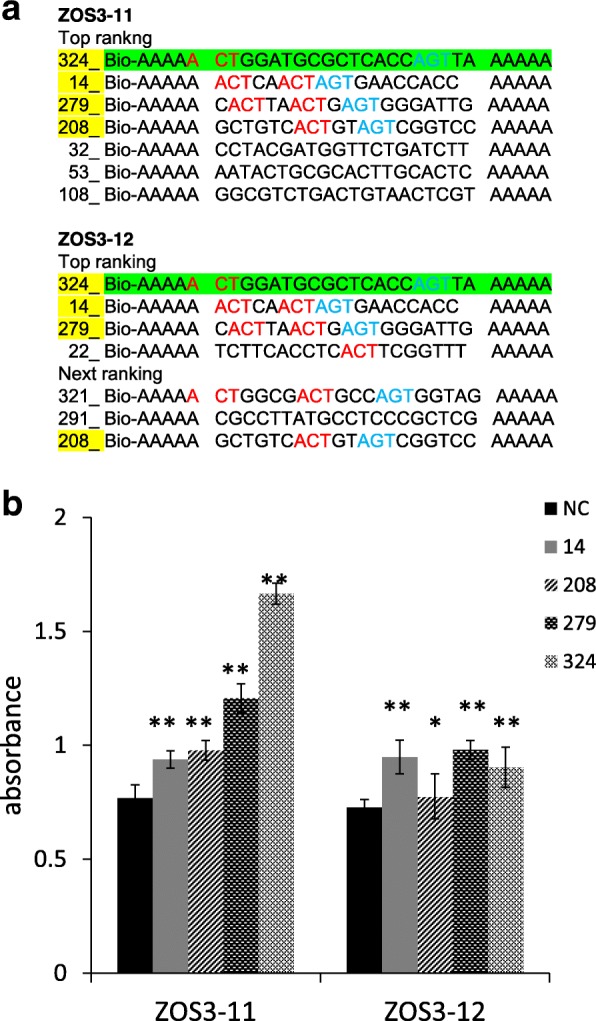
(**a**) Positively bound probes in DPI-ELISA method. The colored letters show the ACT and AGT sequences present in the probes. The probe highlighted in green colour showed highest absorbance. (**b**) Absorbance results of ZOS3–11 and ZOS3–12 binding to the probes [NC (negative control)-AAAAAAGCTTCGCGCCAGCGGGAAAA, 14- AAAAAAACTCAACTA GTGAACCACCAAAA, 208- AAAAAAGCTGTCACTGTAGTCGGTCCAAAA, 279- AAA AAACACTTAACTGAGTGGGATTGAAAA, 324- AAAAAACTGGATGCGCTCACCAGTT AAAAA]. Error bars show the standard deviation value. The asterisk shows a significant difference between the probes with NC (*, *P* < 0.05; **, *P* < 0.01) by Student’s t-test

To prove the DNA binding ability of ZOS3–11 and ZOS3–12, and to get an idea of their specific DNA-binding sites, DNA-Protein-Interaction (DPI)-ELISA technology was adapted [35]. The expression of purified recombinant histidine-tagged ZOS3–11 and ZOS3–12 was confirmed with SDS PAGE and western blot (Additional file 1a), the probes which were positively bound by the protein in DPI-ELISA assay are shown in Fig. 3a. Graphs were plotted based on the absorbance on the positive well in the y-axis and the positive probe number in the x-axis (sequences shown in the figure legend) (Fig. 3b). Both proteins showed similar result by binding to same probes and highest affinity was shown for the sequence where ACT and AGT were separated by 13 bp (Fig. 3a). This result was similar to three petunia ZPT2-related proteins, ZPT2–1, ZPT2–2, and ZPT2–4, binding AGT core sequence separated by 10 bp [36], the wheat WZF1 protein interacting with tandem copies of a CACTC sequence known as ACT box; [37]. As the ACT box has the AGT core sequence in the reverse orientation, it shows the importance of A(G/C)T for the binding of the ZOS3–11 and ZOS3–12. To confirm the importance of A(G/C)T repeat sequences within the probe sequences for binding ZOS3–11 and ZOS3–12, the probe which showed the highest positive result for both the proteins was selected (Probe number 324 having sequence Bio-AAAAAACTGGATGCGCTCACCAGTAAAAA) which contained ACT and AGT sequence separated by 13 nucleotides. Different probes were created by base substitution with mutating the ACT, AGT and both and also the nucleotides in between and the DPI-ELISA assay was repeated to show alteration in the binding capacity of the protein, which confirms specific binding of the fusion protein to ACT and AGT and these sequences seem to be the core target site of ZOS3–11 and ZOS3–12 and also the sequences between ACT and AGT might also influence on the binding capacity (Additional file 1 b, c).

The experiments described above revealed that ZOS3–11 and ZOS3–12 are nuclear localized. Furthermore, it could be confirmed that they can bind to DNA like their homologs from other plant species and may also act as transcription factors. But therefore, more information must be gained in future to know more about its function and downstream targets.

### Overexpression of *OsJAZ8* and ***OsJAZ8ΔC*** shows better adaptation in different stress condition

In the next step, we cloned the promoters of these both transcription factors and fused it with the full-length cDNA of *OsJAZ8* and a version in which the C-terminus was truncated, respectively. The generated constructs ZOS3–11::JAZ8 and ZOS3–12::JAZ8 containing *OsJAZ8* and ZOS3–11::JAZ8ΔC and ZOS3–12::JAZ8ΔC containing *OsJAZ8ΔC* under the control of salt-inducible promoters of *ZOS3–11* and *ZOS3–12* were transformed into BY-2 cells and the presence of the transgene was confirmed by PCR (Additional file 1). The stable cell lines were used for further experiments. To detect potential effects of salt and MeJA on the WT (wild-type) and the transgenic BY-2 cell lines, these were monitored by quantitative phenotyping, using cell viability, packed cell volume, cell elongation, and average cell doubling time as parameters. There were no morphological differences between the transgenic and the WT BY-2 cells under normal growth conditions and under salt stress, ZOS3–12::JAZ8 and ZOS12::JAZ8ΔC did not show any significant difference compared to WT (data not shown).

**Fig. 4:**
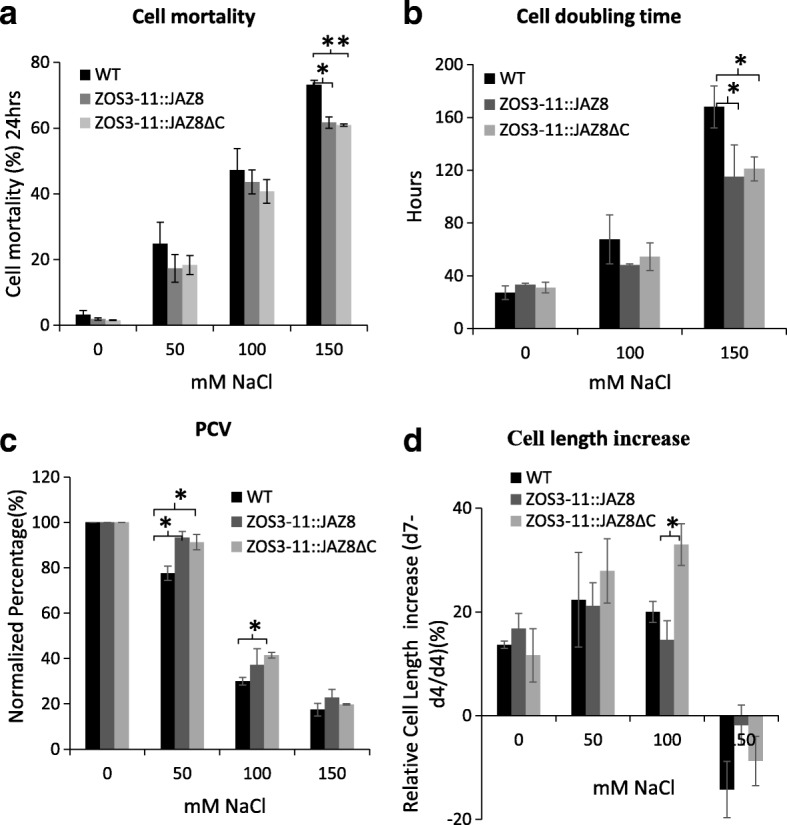
Measurement of different parameters of WT, ZOS3–11::JAZ8 and ZOS11::JAZ8ΔC cells treated with a series of salt concentration of 50 mM, 100 mM and 150 mM. (**a**) Cell mortality percentage (**b**) Average cell cycle doubling time (**c**) Packed cell volume (PCV) (**d**) Relative cell length increase in the stationary phase (d4 = day 4, d7 = day 7). Data represent average of three biological replicates. Error bars show the standard error value. The asterisk shows a significant difference compared with wild type (*, *P* < 0.05; **, *P* < 0.01) by Student’s t-test

When under salt stress, ZOS3–11::JAZ8 and ZOS3–11::JAZ8ΔC showed 10% reduction in cell mortality rate at 150 mM salt compared to the WT, but no significant difference at 50 and 100 mM salt stress (Fig. 4a). The cell cycle duration gives an estimation of time taken to double the number of cells and it can be detected from the cell density taken in time course manner based on the model of exponential growth. The results clearly show a longer time taken for WT cells to divide compared to the transgenic cell lines at 100 and 150 mM salt stress (Fig. 4b). Approximately, 20 and 10% increase in packed cell volume (biomass) at 50 and 100 mM salt stress compared to the WT was observed (Fig. 4c). Cell elongation happens during the stationary phase of the cell cycle. To determine whether salt affects the cell elongation process, the length of the cells treated with different concentration of salt was measured during the start (fourth day) and end of the stationary phase (seventh day) and relative increase of cell length was calculated. All the cell line showed an increase in cell length at 50 mM then gradually decreased with increase in salt concentration, with the exception where a considerable increase (13%) in cell length was observed in case of ZOS3–11::JAZ8ΔC at 100 mM salt (Fig. 4d).

**Fig. 5:**
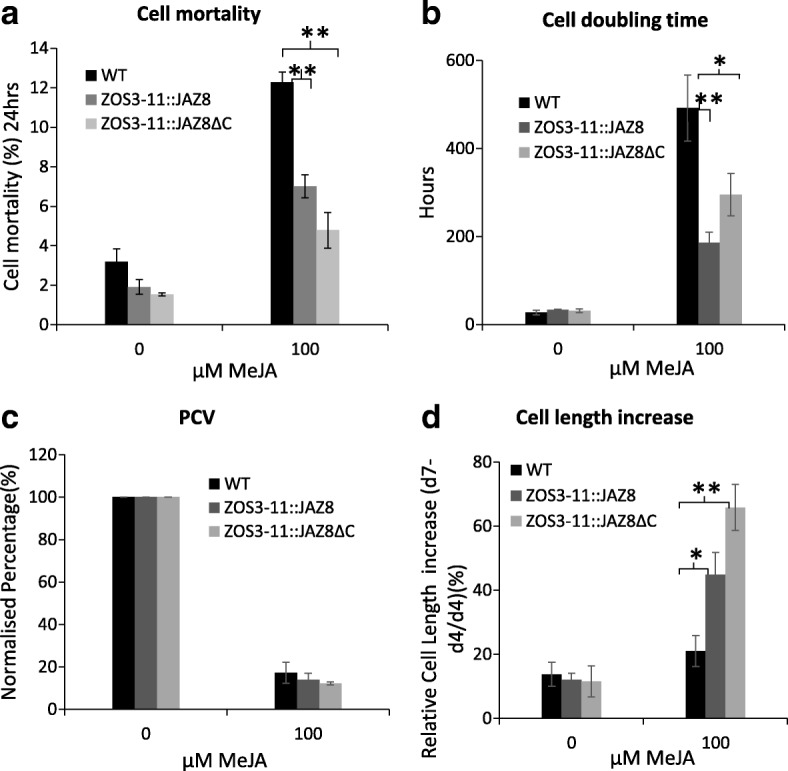
Measurement of different parameters of WT, ZOS3–11::JAZ8 and ZOS11::JAZ8ΔC cells treated with 100 μM of MeJA. (**a**) Cell mortality percentage (**b**) Average cell cycle doubling time (**c**) Packed cell volume (PCV) (**d**) Relative cell length increase in the stationary phase (d4 = day 4, d7 = day 7). Data represent average of three biological replicates. The asterisk shows a significant difference compared with wild type (*, *P* < 0.05; **, *P* < 0.01) by Student’s t-test

Since the expression of ZOS3–11 is regulated by jasmonate (Fig. 2a), the experiments performed with salt were repeated with 100 μM MeJA. ZOS3–11::JAZ8 and ZOS3–11::JAZ8ΔC showed a significant reduction in cell mortality rate (Fig. 5a) and cell doubling time (Fig. 5b) compared to the WT. Cell density decreased tremendously in all the cell lines and transgenic cell lines showed no significant difference when compared with WT (Fig. 5c). Surprisingly, there was 44 and 65% increase in cell length in ZOS3–11::JAZ8 and ZOS3–11::JAZ8ΔC as compared to 20% in WT in the stationary phase (Fig. 5d). Since auxin is shown to be a mediator in cell elongation, effect of auxin on cell elongation is studied which is shown in the section below.

To confirm the overexpression of *OsJAZ8* in ZOS3–11::JAZ8 and *OsJAZ8ΔC* in ZOS3–11::JAZ8ΔC, RNA was extracted from the cell culture at an interval of 1 h, 6 h and 24 h after treatment with 150 mM NaCl and 100 μM MeJA and compared the gene expression patterns. There was no detectable increased expression with salt treatment but highly induced expression was found in response to MeJA in the ZOS3–11::JAZ8 and ZOS3–11::JAZ8ΔC. The highest expression level was detected at 6 h, approximately 15-fold in case of *OsJAZ8* and 17-fold for *OsJAZ8ΔC* respectively and was decreased at 24 h (Additional file 1). Dual-luciferase assay was used to confirm the activity of the promoter ZOS3–11 which was induced by MeJA (2-fold) but not by NaCl (Additional file 1).

### Auxin responsiveness in the transgenic BY-2 cell lines ZOS3–11::JAZ8 and ZOS3–11::JAZ8ΔC

**Fig. 6:**
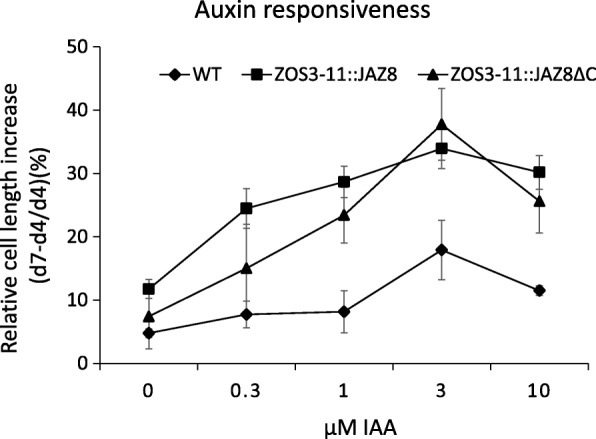
Auxin response in WT and jasmonate insensitive BY-2 transgenic cell lines (ZOS3–11::JAZ8 and ZOS3–11::JAZ8ΔC). The values represent the relative cell length increase of 7 d old cells after IAA treatment with 4 d old cells before IAA treatment. Data represent average of three biological replicates. Error bars show the standard error value

To understand the growth stimulation in the transgenic cell lines compared to the WT, we assayed the auxin sensitivity and responsiveness of cell length increment (Fig. 6). When the dose-response curve of relative cell length increase was determined, the amplitude of the response was found to be dramatically elevated along with an increase in auxin concentration. This was especially impressive when growth in the WT was less induced, whereas it proceeded at almost the maximal velocity in the transgenic cell lines especially ZOS3–11::JAZ8ΔC. In contrast, the threshold and the maximum of the curve were reached at the same concentrations of auxin (3 μM IAA) as in the WT. Thus, there are no indications for an increase of auxin sensitivity, but the transgenic cell lines show amplified responsiveness in response to auxin.

### Expression of jasmonate dependent genes in the transgenic rice lines in response to salt stress

*OsJAZ8* and *OsJAZ8ΔC*-expressing rice plants under the control of salt inducible promoter ZOS3–11 were generated by *Agrobacterium*-mediated transformation. Two independent lines (Line 1 = L1 and Line 2 = L2) of the second or third generation after transformation were used for further experiments.

**Fig. 7:**
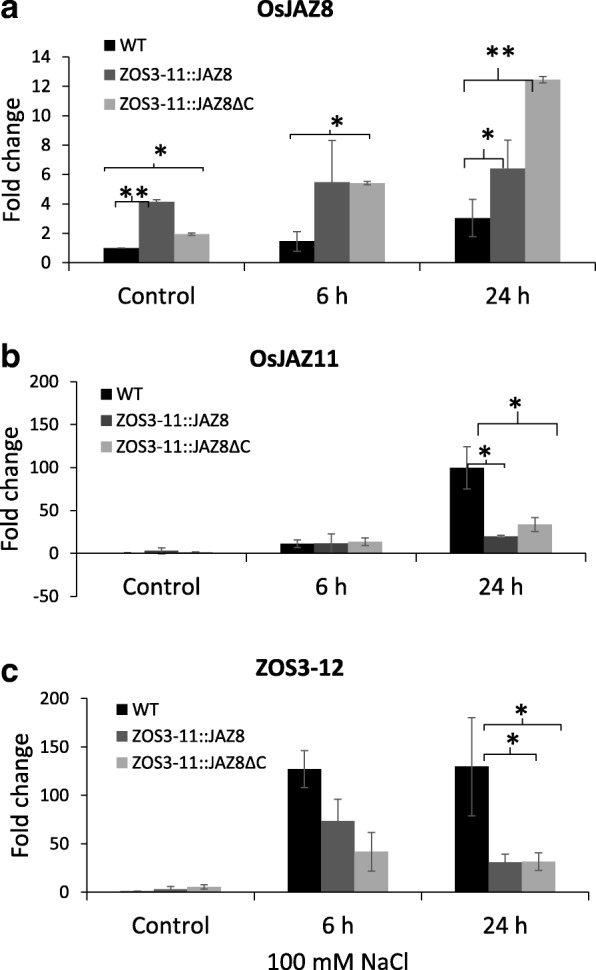
Relative gene expression of (**a**)*OsJAZ8,* (**b**) *OsJAZ11,* (**c**) *ZOS3–12* in WT, transgenic rice leaves (ZOS3–11::JAZ8 (L1) and ZOS11::JAZ8ΔC (L1)) in response to 100 mM salt at 6 h and 24 h relative to WT control. Data represent average of two biological replicates with three technical replicates. The relative amount of transcript was determined by normalization of the housekeeping genes OsUBQ10 and OsUBQ5. Error bars show the standard error value. The asterisk shows a significant difference compared with wild type (*, *P* < 0.05; **, *P* < 0.01) by Student’s t-test

*JAZ*s are JA-responsive genes induced rapidly under salt stress [38]. In order to test whether this response is altered in the transgenic lines, we measured the expression levels of *OsJAZ11* and *ZOS3–12*, which were found to be salt-inducible in a JA-dependent manner in this study. Therefore hydroponic 10-d-old plants were subjected to salt stress with 100 mM NaCl for 6 h and 24 h. The relative transcript levels of *OsJAZ*8, Os*JAZ11,* and *ZOS3–12* were quantified in the leaves by real-time PCR assays and compared to the mock control condition. The relative expression levels of the *OsJAZ8* with WT control were significantly greater in the transgenic lines than in the WT. On average, the transgenic lines showed 5- to 10-fold the expression of WT control. The transgenic control plants had increased basal level of *OsJAZ8 (*3.8-fold for ZOS3–11::JAZ8 (L1) and 1.6-fold for ZOS3–11::JAZ8ΔC (L1)) compared to the wild type (Fig. 7a). The highest expression level under 100 mM NaCl was detected at 24 h, approximately 7.7-fold in case of ZOS3–11::JAZ8 (L1) and 13-fold for ZOS3–11::JAZ8ΔC (L1) respectively. On the other hand, *OsJAZ11* and *ZOS3–12* transcript levels were increased (120- to 130-fold) at 24 h but the expression levels were significantly less in the transgenic lines (Fig. 7b, c). Hence JA-dependent induction of *ZOS3–12* and *OsJAZ11* in response to salt stress was diminished in the transgenic lines.

### Transgenic rice showed better tolerance to salt stress in the early stages

**Fig. 8:**
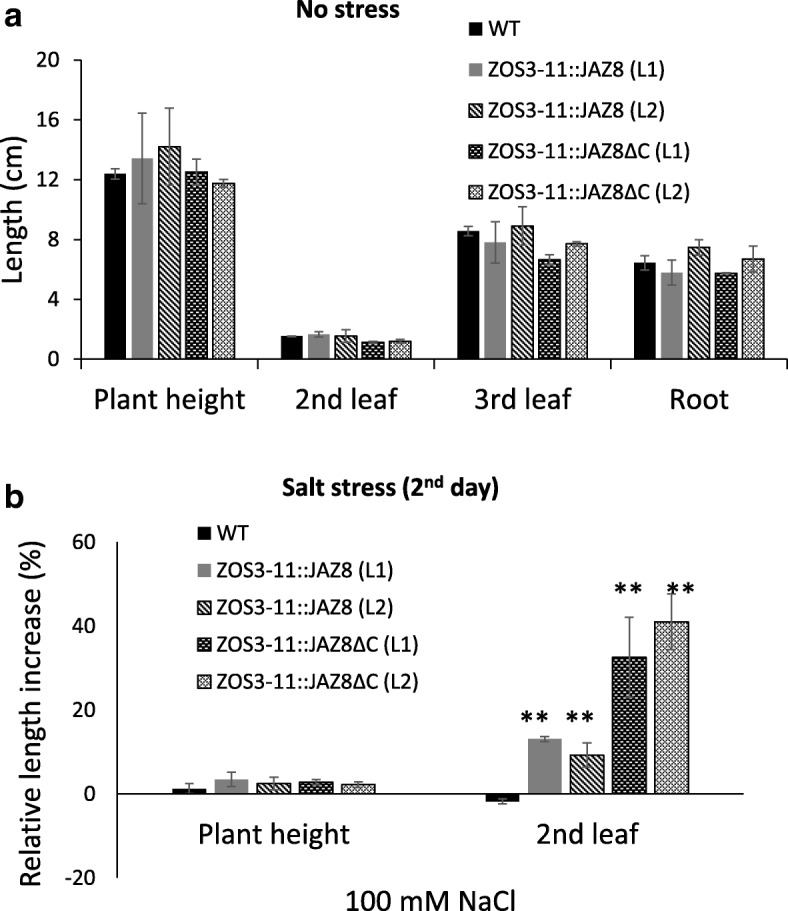
(**a**) Comparison of length of shoot, 2nd leaf, 3rd leaf blade and root of 10-day old WT and transgenic rice ZOS3–11::JAZ8 (L1 & L2), ZOS3–11::JAZ8ΔC (L1 & L2) under no stress condition. (**b**) Relative increase in the length of shoot, 2nd leaf and 2 days after 100 mM salt treatment in 10 day old WT and transgenic rice ZOS3–11::JAZ8(L1 & L2), ZOS3–11::JAZ8ΔC (L1 & L2). Data represent average of three biological replicates. Error bars show the standard error value. The asterisk shows a significant difference compared with wild type (**, *p* < 0.01) by Student’s t-test

The transgenic rice plants did not show morphological differences to the wild-type (WT) in absence of stress (Fig. 8a). However, we noted a higher percentage of grain filling: 12–13% increase in the ZOS3–11::JAZ8ΔC (L1 & L2) was found while ZOS3–11::JAZ8 (L1 & L2) showed 15–20% increase compared to the wild type (Additional file 1). It is yet unclear whether the transgenic lines would show more grain filling under salt stress.

**Fig. 9:**
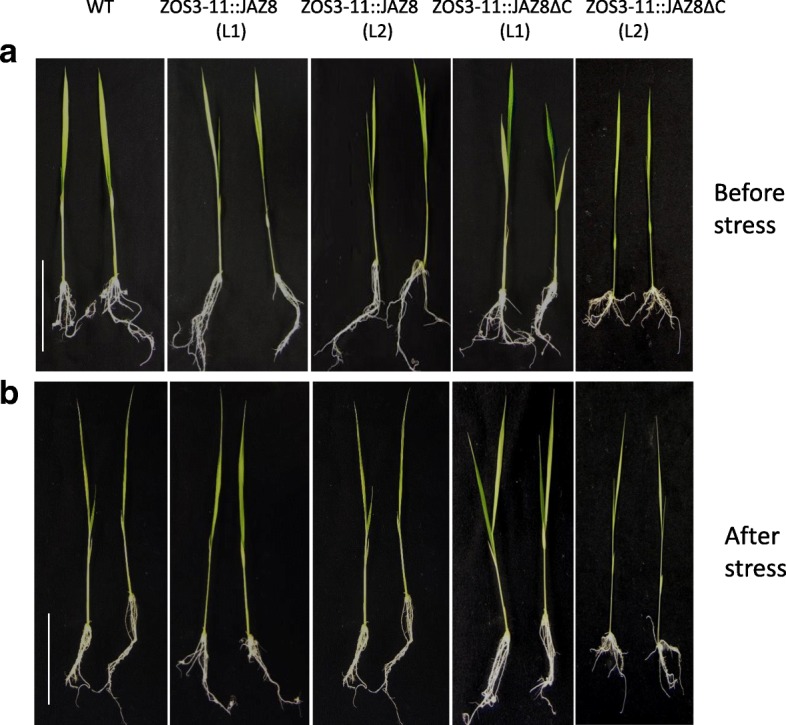
Observation of rice plants (WT, ZOS3–11::JAZ8 (Line 1 = L1 & Line 2 = L2) and ZOS3–11::JAZ8ΔC (Line 1 = L1& Line 2 = L2)) under salt stress. 10 days old rice plants were subjected to 100 mM salt stress (**a**) Plants under control condition (**b**) Plants after 2 days of salt treatment. Scale bar = 5 cm

To check our hypothesis whether dominant suppression of jasmonate will lead to salt stress tolerance, 10 days old seedlings of the WT and the transgenic lines were treated with 100 mM salt and observed for three days. The relative increment in plant height, length of the second leaf (fully developed) and root after two days were calculated. The whole shoot and the root did not show any considerable significant difference. However, the fully developed second leaf of ZOS3–11::JAZ8ΔC showed a significant 50% increase in length compared to the other two (Fig. 8b). After two days, salt treatment clearly showed detrimental effects like leaf rolling and tip burning which was enhanced on the WT leaf compared to the transgenic rice leaf (Fig. 9b). On the third day, the leaves of WT and ZOS3–11::JAZ8 were fully rolled and yellow, while the ZOS3–11::JAZ8ΔC showed tip burning only in one-fourth part of the leaf (Additional file 1). We, therefore, propose that dominant suppression of jasmonate under salt stress improves salinity tolerance warranting future studies. And even though BY-2 transgenic lines ZOS3–12::JAZ8ΔC and ZOS3–12::JAZ8 lines did not show any significant difference in the morphology under salt stress, ZOS3–12::JAZ8ΔC and ZOS3–12::JAZ8 rice lines also will be checked in future as they could show altered responses to salinity stress.

## Discussion

Although the role of jasmonates in salinity adaptation has been widely studied, a straightforward correlation between jasmonate activity and salinity adaptation has not been possible, due to partially contradicting results (reviewed in [6, 9]. One reason for the reported discrepancies may be that often results from different experimental systems are compared that are not really comparable, because the physiological context differs. Differences in temporal patterns of jasmonate accumulation and signalling can lead to qualitatively different responses to salt stress, reviewed in [29]. The concept that transient activation of jasmonate signalling leads to salt stress adaptation was based on correlative evidence – to shift this concept to the analytical level would require that salt-induced jasmonate signalling would be shaped into a transient pattern. This study was mainly intended to develop an innovative strategy to achieve this goal, thus avoiding the disadvantages of a general loss due to deficiency or over-activation of jasmonates and JA signalling leading to salt stress adaptation which makes it different from the studies showing constitutive suppression or other studies where productivity of plants has not been taken into consideration [12]. Making use of the negative feedback loop of JAZ proteins on their own expression, constructs driving full and C-terminal truncated version of *OsJAZ8* genes under the control of salt-induced promoter ZOS3–11 (Additional file 1) were used to suppress jasmonate signalling specifically under high salinity.

The rice Cys2His2 zinc finger transcription factors ZOS3–11 and ZOS3–12 selected based on their close homology with STZ in *Arabidopsis* executing role in salt stress adaptation [22] were jasmonate dependent (Fig. 2) and highly induced under salt stress. The fact that both proteins were localised in the nucleus (Fig. 1) and showed DNA-binding properties (Fig. 3) support the assumption that they are functional in the response to salt stress in rice and act as transcription factors. The binding sequences were identified as A(C/G)T, and in some cases had more than two ACT/AGT. These observations led us to speculate that each ZPT-type zinc-finger domain recognises tandemly repeated A(G/C)T core sequences and that the spacing between each pair of A(G/C)T may be different among the ZPT2-related proteins [34, 36]. To find the target promoters of ZOS3–11 and ZOS3–12, further experiments like ChIP-Seq assay have to be carried out in future, which was clearly beyond the scope of this study, where these promoters were merely used as tools to obtain salt-inducible expression of our construct.

### Suppression of JA signalling in transgenic BY-2 cells leads to salt stress adaptation and JA insensitive phenotypes

In all experiments, several transgenic lines of ZOS3–11::JAZ8 and ZOS3–11::JAZ8ΔC generated from heterologous tobacco BY-2 suspension cell system were used and found to show the same results. Under higher salt concentration of 100 and 150 mM salt stress, the transgenic cells adapted more efficiently compared to the WT BY-2 cells (Fig. 4a, b and c). These observations support a model, where OsJAZ8 and OsJAZ8ΔC, even at low levels of expression (Additional file 1), may help the cells to modulate temporal patterns and shift the negative effect of jasmonate in response to high salinity towards adaptation. This was in agreement with studies where constrained JA accumulation and signalling correlated with a precondition to escape salinity-induced cell death and to activate salinity adaptation [29]. Eventually, the transgenic cell lines under salt and MeJA treatment clearly showed a jasmonate insensitive phenotype evident from an increase in cell length in the stationary phase especially in ZOS3–11::JAZ8ΔC (Figs. 4d and 5d). These properties are consistent with *Arabidopsis* JAZ proteins lacking the C-terminal Jas region which were resistant to COI1-dependent degradation after jasmonate treatment, and that dominantly showed jasmonate-resistant phenotypes, such as resistance to JA-induced inhibition to root elongation [14, 16, 39, 40]. These discrepancies in the response to salt and MeJA treatment correlated with the expression pattern of the *OsJAZ8*, which was highly expressed by MeJA, but not by salt in transgenic lines (Additional file 1). And this difference in *OsJAZ8* expression correlates in turn with the observation that the activity of the *ZOS3–11* promoter is higher with MeJA as compared to salt stress (Additional file 1). Cell elongation of the transgenic BY-2 cell lines ZOS3–11::JAZ8 and ZOS3–11::JAZ8ΔC under MeJA (Fig. 5d) can be correlated with previous studies which reported that treatment with MeJA promotes the production of genes involved in IAA synthesis [41] leading to increased IAA level and also involvement of COI1-dependent signalling pathway in regulation of these genes [42]. Thus, suppression of jasmonate signalling in the transgenic lines may confer increases in IAA activity leading to cell elongation. Conversely, when dose-response relations for auxin-dependent cell expansion were recorded, the transgenic BY-2 cell lines showed an increased responsiveness to auxin manifest as a higher amplitude of the elongation response, and the effect was elevated in the ZOS3–11::JAZ8ΔC where the jasmonate signalling should be dominantly suppressed. These results match previous data in the jasmonate biosynthesis mutant *hebiba* where the absence of JA was linked with an enhanced responsiveness of coleoptile elongation to exogenous IAA [30, 43], or altered coleoptile bending in response to gravity [44]. This phenomenon can be explained by considering that on the level of hormone perception and signalling, both IAA and JA-Ile share the same components like SCF E3-ligases, but also the upstream regulator AUXIN RESISTANT1 (AXR1). Mutations in these components, therefore, cause impaired responses to both hormones [45, 46].

### Transgenic rice showed salt stress adaptation in the early stages

While the observations in tobacco BY-2 supported the notion that the construct driving a transient jasmonate signature in fact improved salt tolerance, the evidence from the homologous system, rice, was still warranted. Various reports demonstrate that constitutive JA-deficiency in case of rice [30] and *Arabidopsis* [47–49] and expression of *AtJAZ1ΔJas* and *AtJAZ10.4* which lacks Jas domain, resulted in male sterility [16, 39] By inducing a (salt-dependent) transient activation of jasmonate signalling by constructing the transgenic rice plants ZOS3–11::JAZ8 and ZOS3–11::JAZ8ΔC where the expression of *OsJAZ8* and *OsJAZ8ΔC* was under the control of salt-inducible promoters *ZOS3–11* we were able to maintain fertility and even to obtain an increase in the number of filled grains in the absence of salt stress (Additional file 1), although the promoter is responsive to JA, and could potentially be activated during flowering. To what extent these plants can sustain fertility under salt stress conditions, will be subject to future work. However, already now it is clear that under salt stress conditions, the transgenic rice plants ZOS3–11::JAZ8 and ZOS3–11::JAZ8ΔC showed better performance in the vegetative state which was correlated with a higher amount of endogenous *OsJAZ8*, which was detected even under control condition (Fig. 7a). As to be expected the persistence of the adaptation effect was correlated with the degree of persistence for the two engineered transgenes: While the improved salt tolerance produced by ZOS3–11::JAZ8 was seen at early time points, but then vanished such that plants behaved similarly to wild-type in the later days (Fig. 9b), the ZOS3–11::JAZ8ΔC lines showed increased salt tolerance even on the third day. The transient effect seen for the full-length JAZ8 construct may be due to proteolytic degradation at a later time, similar to a previous report, where such a progressive degradation had been seen for overexpression of OsJAZ9 [50]. The dominant repression of jasmonate signalling by the negative activity of OsJAZ8ΔC has been shown previously [31], and our results demonstrate, how this can be utilised to obtain a durable salt tolerance. The suppression of jasmonate signalling and also of jasmonate synthesis is witnessed by the fact that the JA responsive gene JAZ11 (highly expressed under salt stress) and the jasmonate-regulated gene ZOS3–12 show reduced expression (Fig. 7b, c) in the transgenic lines.

## Conclusions

All these observations in our novel approach may be summarised as follows: In response to the activation of the ZOS3–11 promoter in BY-2 cells and in rice, OsJAZ8 and its dominant-negative variant OsJAZ8ΔC were overexpressed in the respective plant tissue to eventually suppress jasmonate signalling and other jasmonate-dependent downstream genes. This controlled repression of jasmonate signalling clearly modulates its temporal signature thereby decreasing its negative effect hence increasing better performance, JA-insensitivity and increased auxin responsiveness in BY-2 cells and early stage enhanced salt stress tolerance in rice. Hormonal quantifications, salt uptake studies and identification of new TFs regulated by OsJAZ8 may provide further evidence and insight into JA regulated salt stress adaption mechanism in rice.

Taken together we tested a strategy to improve salt tolerance of rice by suppressing jasmonate signalling under salinity stress in a proof-of-principle study. In the future, the genetic material developed in this study should be further explored, and additional plants should be generated by the introduction of promoters differing in tissue-specificity and responses to external factors. Especially exploiting salt-inducible promoters that are completely independent of JA would be recommended, as side-effects of JA could be mostly excluded. This would offer an advantage over the current strategy, as we cannot rule out that ZOS3–11::JAZ8 and ZOS3–11::JAZ8ΔC plants would be more sensitive to pathogens or insect attack.

## Methods

### Plant materials

Rice seeds used in this study were either generated in the Botanical Garden of the Karlsruhe Institute of Technology (KIT, Germany) or at CIRAD Montpellier (France) in the respective greenhouse facilities. Tobacco BY-2 suspension cells were cultivated at KIT.

### Localisation of ZOS3–11 and ZOS3–12

RNA was extracted from second leaves of seedlings of *Oryza sativa* L. ssp. japonica cv. Nipponbare raised for 14 days, using the innuPREP Plant RNA Kit (Analytik Jena AG, Jena). The cDNA was synthetised by M-MULV Reverse Transcriptase (New England Biolabs, Frankfurt am Main) from 1 μg of RNA as a template. The coding sequences of *ZOS3–11* (LOC_Os03g32220.1) and *ZOS3–12* (LOC_Os03g32230.1) were amplified using specific primers designed by Primer 3 online software (http://www.bioinformatics.nl/cgi-bin/primer3plus/primer3plus.cgi, last accessed 21 September 2017). The coding regions, extended by the Gateway® attB sites were amplified and inserted into the entry vector pDONR™/Zeo (Life Technologies, Germany) using the PCR conditions given in the Additional file 1, and the amplicons then cloned into the destination vector p2GWF7 [51] yielding a fusion with GFP placed at the C-terminus by Gateway®-Cloning technology (Invitrogen Corporation, Paisley, UK). The resulting plasmids p2GWF7ZOS3–11 und p2GWF7ZOS3–12 were verified by sequencing, and then used to perform biolistic transformation into etiolated coleoptiles raised for four days as described by [52].

### Determination of the DNA target motif for ZOS3–11 and ZOS3–12 binding

To identify potential DNA target motifs for the binding of the ZOS3 transcription factors, full-length coding sequences for *ZOS3–11* and *ZOS3–12* without stop codon were cloned into the Gateway®-pET-DEST42 vector (Life Technologies, Germany) containing a N-terminal His-tag via Gateway®-Cloning technology (Invitrogen Corporation, Paisley, UK) and transformed into the *E. coli* expression strain BL21/RIL (DE3) (Stratagene, Germany). As negative control, a *pET-DEST42-empty* vector without *ccDB* cassette was used [35]. The positively transformed colonies were picked and incubated in 5 ml culture flasks containing LB medium supplemented with ampicillin (100 μg ml^− 1^) overnight. These pre-cultures were then transferred to 500 ml LB medium and induced by the addition of 500 μM of Isopropyl β-D-1-thiogalactopyranoside (IPTG) for 2 h with a start OD_600_ of 0.1 and grown to an OD_600_ of 0.6–0.8 at 37 °C under shaking with 180 rpm. The protein extraction and DPI-ELISA protocol were followed as described in [35]. Extracted proteins were separated on 10% (*w*/*v*) polyacrylamide gels by SDS-PAGE and subsequently probed and analyzed by Western blotting using a monoclonal mouse anti-histidine antibody 1:2000 (Penta His Antibody, BSA-free, Qiagen, 1:2000 diluted in TBS buffer) as primary antibody, and visualization by a secondary antibody (Anti-mouse IgG, alkaline phosphatase-conjugated (Sigma, St. Louis, USA), 1:50000 diluted in TBS buffer) with reference to the Color Prestained Protein standard (Broad Range 11–245 kDa, New England Biolabs, Frankfurt, Germany) as protein ladder.

### Quantification of steady-state transcript levels

RNA was extracted from rice leaves and tobacco BY-2 cells using the InnuPrep plant RNA kit (Analytik Jena) according to the instructions of the manufacturer. For rice, a small amount of leaf material (100 mg) was shock-frozen in liquid nitrogen and then ground to a powder (Tissue Lyzer, Qiagen, Hilden, Germany), in case of tobacco BY-2 cells, 2 ml of cell suspension were drained from liquid medium using filter paper, transferred to a 2-ml reaction tube (Eppendorf, Hamburg) before freezing in liquid nitrogen and grinding. The extracted RNA was reversely transcribed into cDNA by M-MULV Reverse Transcriptase (New England Biolabs, Frankfurt am Main) using 1 μg RNA as a template. Real-time (qPCR) was performed with the CFX96 Touch™ Real-Time PCR Detection System from Bio-Rad Laboratories GmbH (Munich) using a SYBR Green dye protocol. Transcript levels between different samples were compared using the ΔΔCt method was used [53]. EF-1α (LOC_Os03g08010), UBQ5 (LOC_Os01g22490), and UBQ10 (LOC_Os03g13170) and UBQ5 (LOC_Os01g22490) were used as endogenous controls for normalisation in case of rice, in case of tobacco BY-2 cells, NtGADPH (NM_001325431) served as housekeeping gene. At least three biological replicates were performed for each treatment, for the transgenic rice plants, three to five individuals from two independent transformants were used. Three technical replicates were conducted from each biological replication. Details for primers and PCR conditions are given in (Additional file 1).

### Construction of OsJAZ8/OsJAZ8ΔC overexpressing vectors under the influence of jasmonate dependent promoters

A full-length cDNA of *OsJAZ8* (LOC_Os09g26780) (amino acid 233) and Jas domain truncated (OsJAZ8ΔC; amino acids 1–176), according to [31] was ligated into destination vector pMDC107 [54] in-frame replacing N-terminus GFP to create recombinant plasmid pMDC107-OsJAZ8/OsJAZ8ΔC for expressing the fusion protein. (Primers in Additional file 1). The promoter regions (2000–3000 bp upstream of the start codon) of *ZOS3–11* (LOC_Os03g32220.1) (2930 bp) and *ZOS3–12* (LOC_Os03g32230.1) (2092 bp) were extracted and amplified from the isolated genomic DNA using specific primers for GATEWAY cloning (see Additional file 1) designed by Primer 3plus online software. For PCR, the enzyme Q5® High-Fidelity DNA polymerase (New England Biolabs, Frankfurt am Main) was used. Promoter regions were inserted into the destination vector pMDC107-OsJAZ8/OsJAZ8ΔC using the Gateway®-Cloning technology (Invitrogen Corporation, Paisley, UK). Positive plasmids were confirmed by restriction analysis and further verified by sequencing (GATC Biotech, Cologne, Germany). verified by DNA sequencing (GATC Biotech, Cologne, Germany).

### Transformation of BY-2 tobacco cells

Tobacco BY-2 (*Nicotiana tabacum* L. cv BY-2) suspension cultures [55] were used for the transformation. Different stable transgenic tobacco BY-2 cell lines having ZOS3–11::JAZ8, ZOS3–11::JAZ8ΔC, ZOS3–12::JAZ8 and ZOS3–12::JAZ8ΔC were obtained by electroporation of *Agrobacterium tumefaciens* LBA4404 (Invitrogen) based method developed by [56] with several modifications for better performance. The transgenic calli were selected on a medium containing 40 mg l^− 1^ hygromycin. The presence of the inserts was confirmed using PCR amplification (Additional file 1). After approximately 3 weeks incubation, the positive calli were transferred onto fresh MS agar plates (with corresponding antibiotics and cefotaxime) for further growth and a suspension culture was then established from the pooled calli after enough of them had grown into appropriate sizes. The resulting lines, thus, represent a population of different transgenic cell strains.

### Cell cultures

1.0–1.5 Ml of tobacco BY-2 WT and the transgenic suspension cultures cells in stationary phase were subcultivated into 30 ml fresh liquid medium containing 4.3 g/l Murashige and Skoog salts (Duchefa Biochemie, Haarlem, the Netherlands), 30 g l^− 1^ sucrose, 200 mg l^− 1^ KH_2_PO_4_, 100 mg l^− 1^ inositol, 1 mg l^− 1^ thiamine, and 0.2 mg l^− 1^, 2,4-D, pH 5.8 and incubated at 26 °C in darkness on an orbital shaker (IKA Labortechnik, Staufen, Germany) rotating constantly at 150 rpm. The stock calli were maintained on the solidified MS medium with 0.8% (*w*/*v*) agar (Roth, Karlsruhe, Germany) and sub-cultivated monthly

Salt-stress (50–150 mM NaCl) and MeJA (100 μM), were administered at the time of subcultivation and auxin (indole acetic acid, 0.3–10 μM) on the fourth day after subculture.

### Determination of cell mortality and packed cell volume, effect on cell length and cell cycle

Cell mortality was quantified using Evans blue dye exclusion assay [57]. Aliquots (0.5 ml) taken after 24 h of treatment were incubated into 2.5% Evans Blue (w/v) (Sigma-Aldrich) for 3 min. Dead cells were counted using a brightfield microscope after rinsing three times with fresh distilled water. Percentage of dead cells was calculated and plotted. 1000 cells were counted for each experiment. The biomass was calculated by measuring the packed cell volume (PCV) [58] day four after stress treatment. Cell length was measured using the length measurement function of the AxioVision software according to [59]. The plotted data point represents the relative increase in the cell length from the fourth to seventh day from at least 500 individual cells. To compare the effect of salt (100 mM and 150 mM) and MeJA (100 μM) on doubling time of cell cycle, cells were collected from day 0 to day 3 and cell density was estimated by a hematocytometer (Fuchs-Rosenthal), using an exponential model for proliferation (Nt = N0.e^kt^ with Nt cell density at time point t, N0 cell density at inoculation, and k the time constant). The starting number (N0) was quantified just after sub-cultivation to set the reference. Three independent experimental series were conducted for each experiment.

### Assay of promoter activity by dual-luciferase assay system

The entry vector containing promoter region of *ZOS3–11* (LOC_Os03g32220.1) (2930 bp) (mentioned above) was ligated into the luciferase vector pLUC [60] using the GATEWAY®-Cloning technology (Invitrogen Corporation, Paisley, UK) and verified by DNA sequencing (GATC Biotech, Cologne, Germany). A well-established dual-luciferase system based on transient transformation [61] was employed to measure promoter activation. Three-day-old cells from WT BY-2 cells placed on solid MS medium (0.8% Danish agar) were used to check the activity of *ZOS3–11* promoters which was measured in response to elicitation by either 100 mM NaCl or 100 μM MeJA, or a related solvent without elicitor (as control) administered 24 h after transfection. Three independent experimental series were performed.

### Transformation of Rice

The *japonica* variety *Kitaake* of rice (*Oryza sativa* L.) was used for this study. The transgenic lines were generated at the Centre de Cooperation Internationale en Recherche Agronomique pour le Developpement (CIRAD, Montpellier, France) according to the method mentioned in [62]. T2 generation seeds of two different lines of transgenic plants were used for experiments. ZOS3–11::JAZ8 (Line 1 10.4 and Line 2 19.5(1)), ZOS3–11::JAZ8ΔC (Line 1 4.1and Line 2 1.1). The seed sterilization and growth conditions were according to [10]. After 10 days, plantlets were transferred to custom-made sterilized floating racks and moved to a glass container containing double-distilled water and placed in a growth chamber (14-h-light/10-h-dark cycles) at 25 °C. After two days in the hydroponic condition in water, they were used for the salt stress treatments and double-distilled water was used as control. For phenotyping, seedlings were treated with 100 mM NaCl solution, length of the whole shoot, second leaf and root were measured before and second day after salt treatment and photographed. Percentage increment of the length was calculated. Results shown are from three independent experiments.

## Additional file

Additional file 1: **Figure S1.** The phylogenetic tree of selected rice stress responsive C2H2-type zinc finger proteins and STZ/ZAT10 [63, 64]. **Figure S2.** Multiple sequence alignment of amino acid sequences of rice stress-responsive C2H2-type zinc finger proteins with STZ/ZAT10. **Figure S3.** Localization study of ZOS3–11 and ZOS3–12 in BY2 cells. **Figure S4.** DNA-binding capacity of ZOS3–11 and ZOS3–12. **Figure S5.** Confirmation of T-DNA inserts in BY-2 transgenic cell lines by PCR. **Figure S6.** Relative gene expression of *OsJAZ8* in transgenic BY-2 lines ZOS3–11::JAZ8 and *OsJAZ8ΔC* in ZOS3–11::JAZ8ΔC. **Figure S7.** Dual luciferase assay for measuring ZOS3–11 promoter activity after salt and MeJA treatment. **Figure S8.** Percentage of filled grain in transgenic rice plants. **Figure S9.** Phenotypic observation of third leaf of 10 days old WT and transgenic rice plants subjected to 100 mM salt. **Figure S10.** Schematic diagram the constructs used for transformation. **Table S11.** List of PCR primers with used for Gateway cloning. **Table S12.** List of primers for checking T-DNA inserts. **Table S13.** List of primers used for qPCR. (PPTX 29561 kb)

## Abbreviations

ABA: Abscisic acid
AOC: Allene Oxide Cyclase
AXR1: Auxin resistant 1
bHLH transcription factor: basic Helix-Loop-Helix transcritpion factor
BY-2: Bright Yellow 2
CaMV: Cauliflower Mosaic virus
CHiP: Chromatin immunoprecipitation
COI1: Coronatine insensitive 1
*cpm2*: Coleoptile photomorphogenesis 2
CYP: Cytochrome P450
DPI: DNA-Protein interaction
ELISA: Enzyme-linked immunosorbent assay
IAA: Indole-3-acetic acid
JA: Jasmonic acid
JA-Ile: Jasmonate-isoleucine
JAR1: Jasmonate resistant 1
JAZ: Jasmonate-Zim domain
MeJA: Methyl Jasmonate
Myc transcription factor: Myelocytomastosis transcription factor
PCR: Polymerase chain reaction
RT: Reverse transcription
SCF complex: SKP1-Cullin-F-box protein complex
SKP1: S-phase kinase-associated protein 1
STZ: Salt tolerance Zinc-finger protein
TF: Transcription factor
WZF1: Wheat zinc-finger protein 1
ZAT: Zinc-finger protein of *Arabidopsis thaliana*
ZFP: Zinc-finger protein
ZOS: Zinc-finger protein of *Oryza sativa*
ZPT: Zinc-finger protein
