# Supplementary material for: Salt-inducible expression of OsJAZ8 improves resilience against salt-stress
Source: BMC Plant Biol. 2018 Nov 29;18:311. doi: 10.1186/s12870-018-1521-0 (PMC6267056; doi:10.1186/s12870-018-1521-0)
Supplement: Supplementary file 1 — Figure S1. The phylogenetic tree of selected rice stress responsive C2H2-type zinc finger proteins and STZ/ZAT10 [63, 64]. Figure S2. Multiple sequence alignment of amino acid sequences of rice stress-responsive C2H2-type zinc finger proteins with STZ/ZAT10. Figure S3. Localization study of ZOS3–11 and ZOS3–12 in BY2 cells. Figure S4. DNA-binding capacity of ZOS3–11 and ZOS3–12. Figure S5. Confirmation of T-DNA inserts in BY-2 transgenic cell lines by PCR. Figure S6. Relative gene expression of OsJAZ8 in transgenic BY-2 lines ZOS3–11::JAZ8 and OsJAZ8ΔC in ZOS3–11::JAZ8ΔC. Figure S7. Dual luciferase assay for measuring ZOS3–11 promoter activity after salt and MeJA treatment. Figure S8. Percentage of filled grain in transgenic rice plants. Figure S9. Phenotypic observation of third leaf of 10 days old WT and transgenic rice plants subjected to 100 mM salt. Figure S10. Schematic diagram the constructs used for transformation. Table S11. List of PCR primers with used for Gateway cloning. Table S12. List of primers for checking T-DNA inserts. Table S13. List of primers used for qPCR. (PPTX 29561 kb) [file 12870_2018_1521_MOESM1_ESM.pptx]

## Slide 1
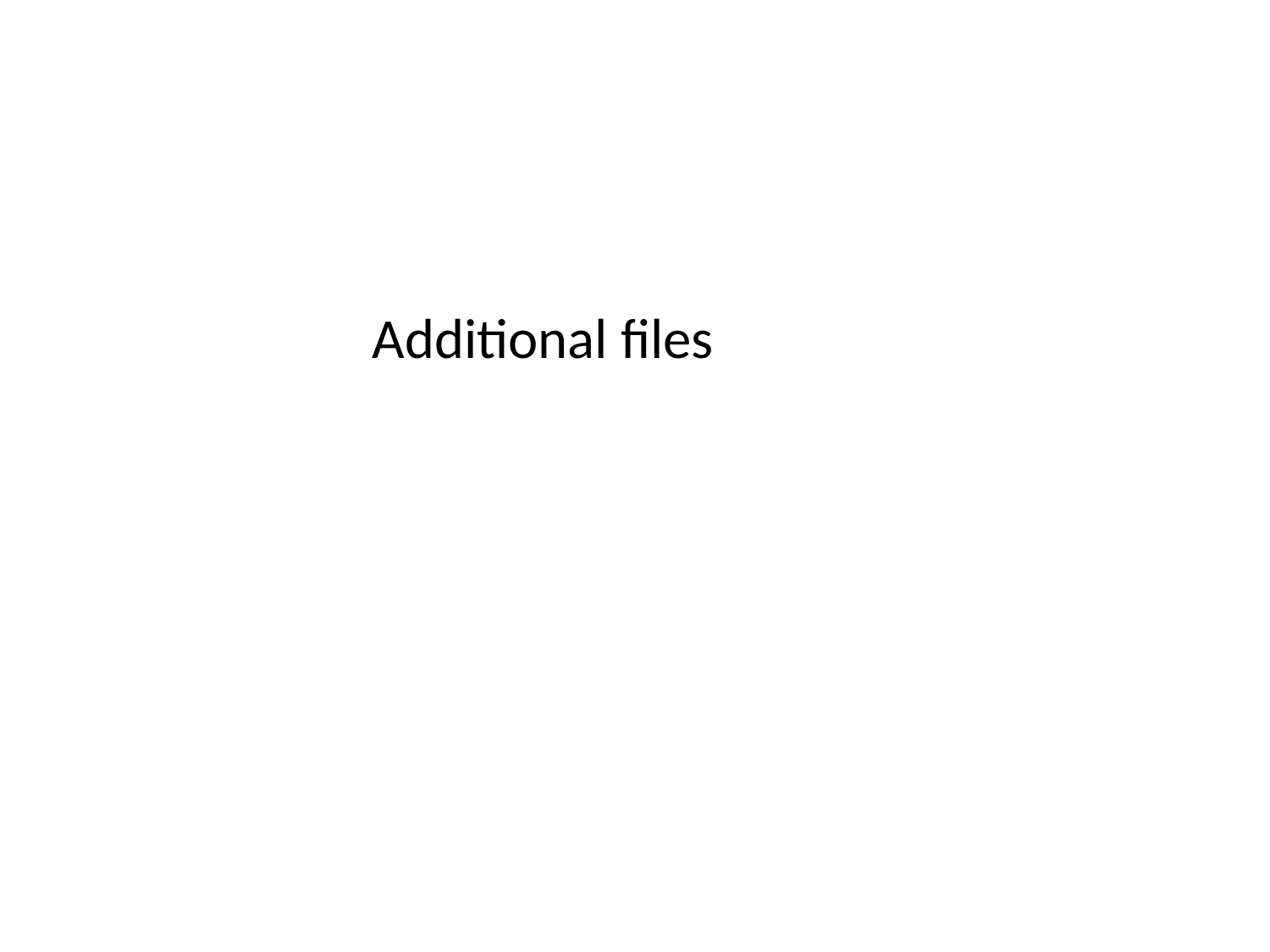

Additional files

## Slide 2
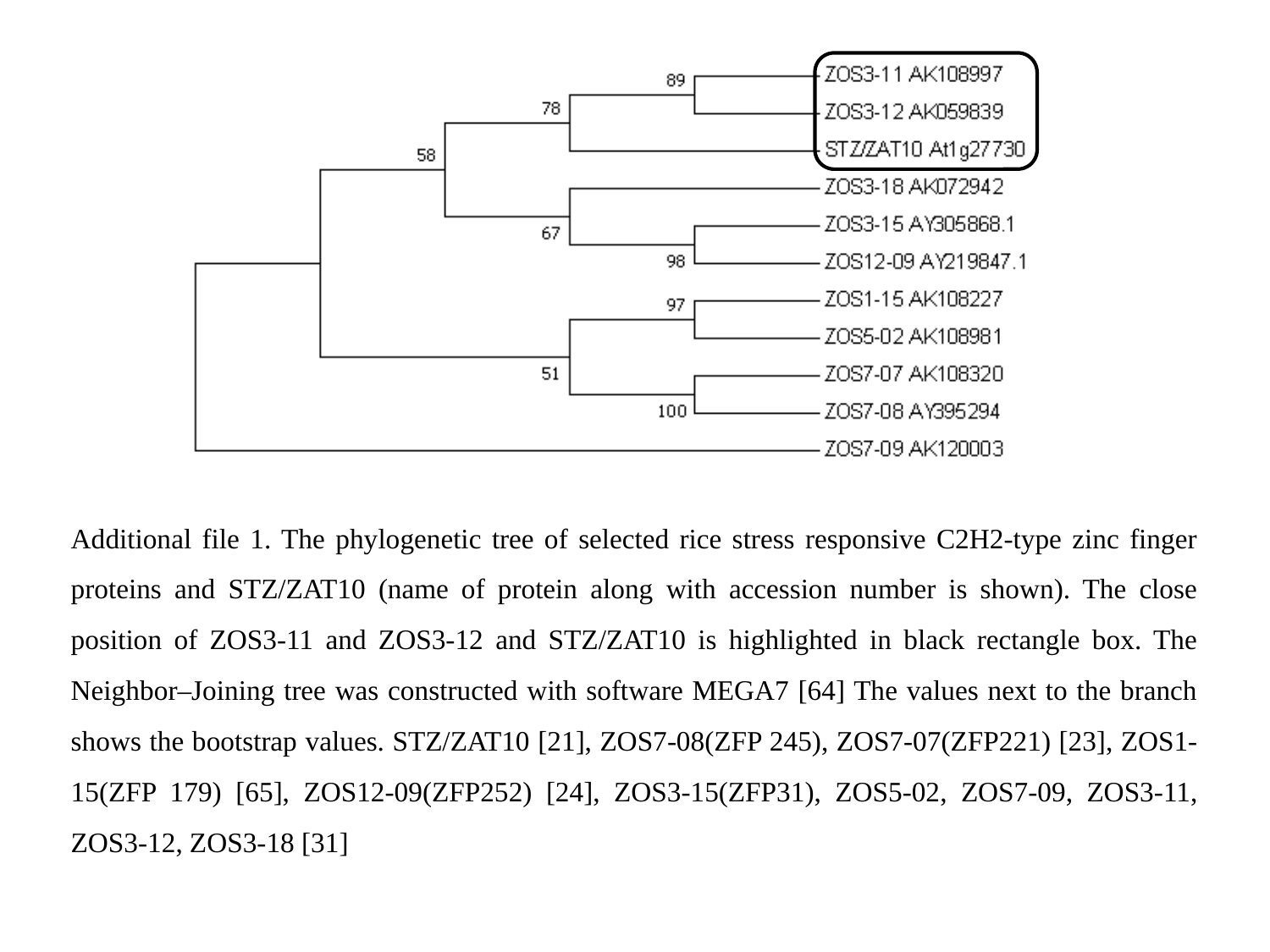

Additional file 1. The phylogenetic tree of selected rice stress responsive C2H2-type zinc finger proteins and STZ/ZAT10 (name of protein along with accession number is shown). The close position of ZOS3-11 and ZOS3-12 and STZ/ZAT10 is highlighted in black rectangle box. The Neighbor–Joining tree was constructed with software MEGA7 [64] The values next to the branch shows the bootstrap values. STZ/ZAT10 [21], ZOS7-08(ZFP 245), ZOS7-07(ZFP221) [23], ZOS1-15(ZFP 179) [65], ZOS12-09(ZFP252) [24], ZOS3-15(ZFP31), ZOS5-02, ZOS7-09, ZOS3-11, ZOS3-12, ZOS3-18 [31]

## Slide 3
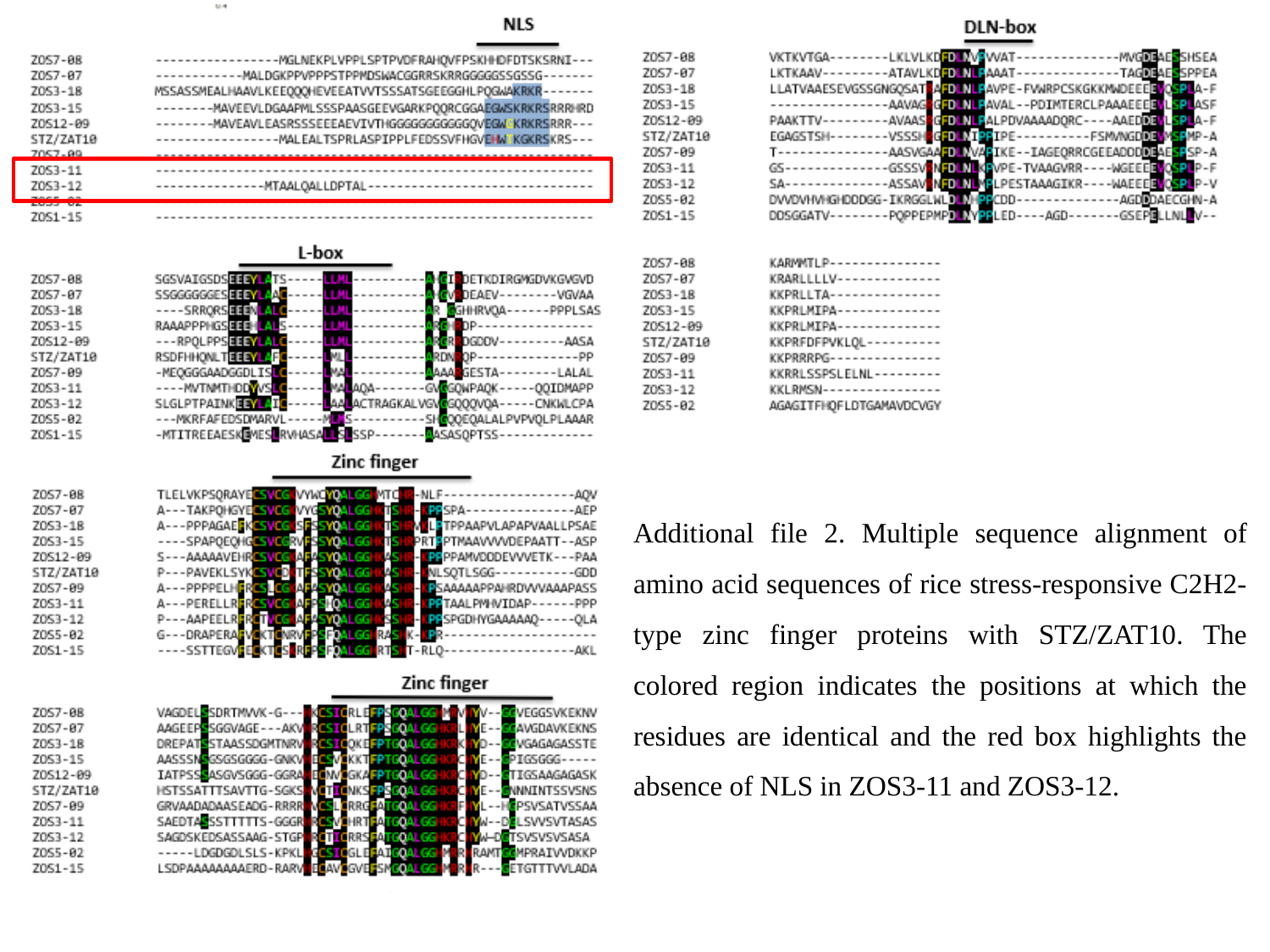

Additional file 2. Multiple sequence alignment of amino acid sequences of rice stress-responsive C2H2-type zinc finger proteins with STZ/ZAT10. The colored region indicates the positions at which the residues are identical and the red box highlights the absence of NLS in ZOS3-11 and ZOS3-12.

## Slide 4
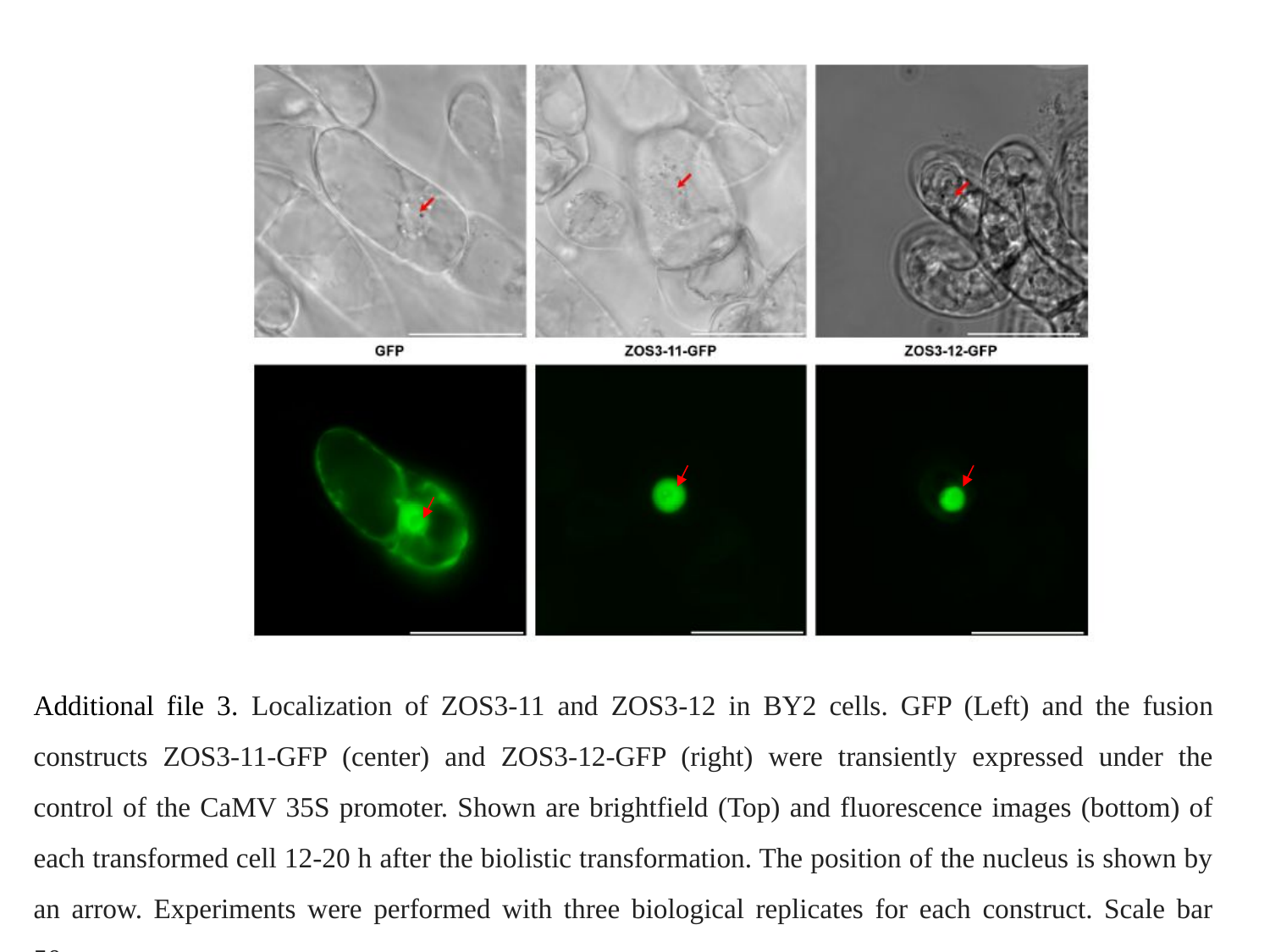

Additional file 3. Localization of ZOS3-11 and ZOS3-12 in BY2 cells. GFP (Left) and the fusion constructs ZOS3-11-GFP (center) and ZOS3-12-GFP (right) were transiently expressed under the control of the CaMV 35S promoter. Shown are brightfield (Top) and fluorescence images (bottom) of each transformed cell 12-20 h after the biolistic transformation. The position of the nucleus is shown by an arrow. Experiments were performed with three biological replicates for each construct. Scale bar 50µm.

## Slide 5
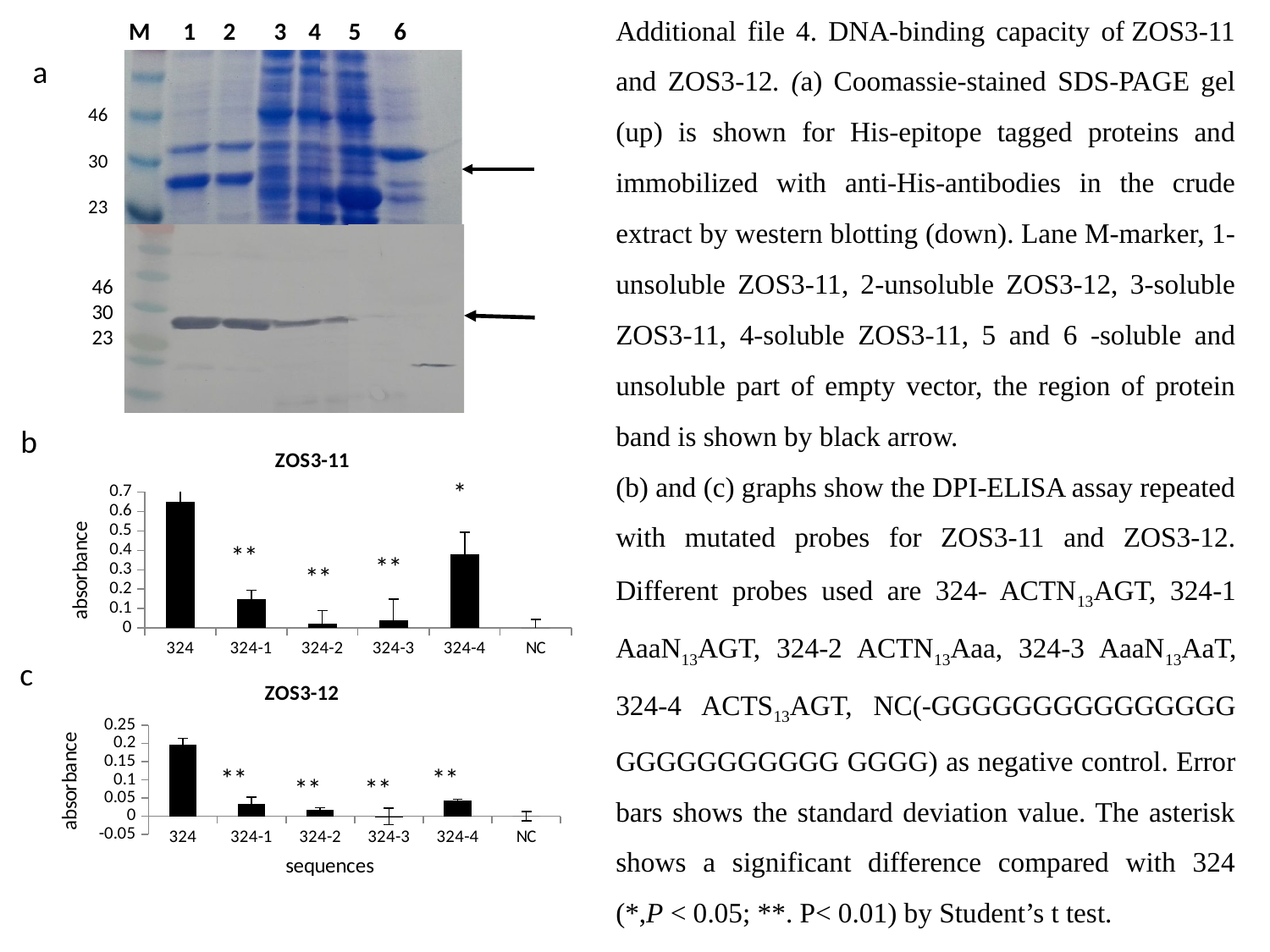

Additional file 4. DNA-binding capacity of ZOS3-11 and ZOS3-12. (a) Coomassie-stained SDS-PAGE gel (up) is shown for His-epitope tagged proteins and immobilized with anti-His-antibodies in the crude extract by western blotting (down). Lane M-marker, 1-unsoluble ZOS3-11, 2-unsoluble ZOS3-12, 3-soluble ZOS3-11, 4-soluble ZOS3-11, 5 and 6 -soluble and unsoluble part of empty vector, the region of protein band is shown by black arrow.
(b) and (c) graphs show the DPI-ELISA assay repeated with mutated probes for ZOS3-11 and ZOS3-12. Different probes used are 324- ACTN13AGT, 324-1 AaaN13AGT, 324-2 ACTN13Aaa, 324-3 AaaN13AaT, 324-4 ACTS13AGT, NC(-GGGGGGGGGGGGGGG GGGGGGGGGGG GGGG) as negative control. Error bars shows the standard deviation value. The asterisk shows a significant difference compared with 324 (*,P < 0.05; **. P< 0.01) by Student’s t test.
 M 1 2 3 4 5 6
46
30
23
46
30
23
a
b
### Chart: ZOS3-11
| Category | |
|---|---|
| 324 | 0.6487500000000006 |
| 324-1 | 0.14825000000000021 |
| 324-2 | 0.022000000000000148 |
| 324-3 | 0.038500000000000124 |
| 324-4 | 0.3795000000000002 |
| NC | 5.551115123125793e-17 |c
### Chart: ZOS3-12
| Category | |
|---|---|
| 324 | 0.197 |
| 324-1 | 0.03475 |
| 324-2 | 0.017999999999999995 |
| 324-3 | -0.0004999999999999989 |
| 324-4 | 0.04324999999999998 |
| NC | 0.0 |

## Slide 6
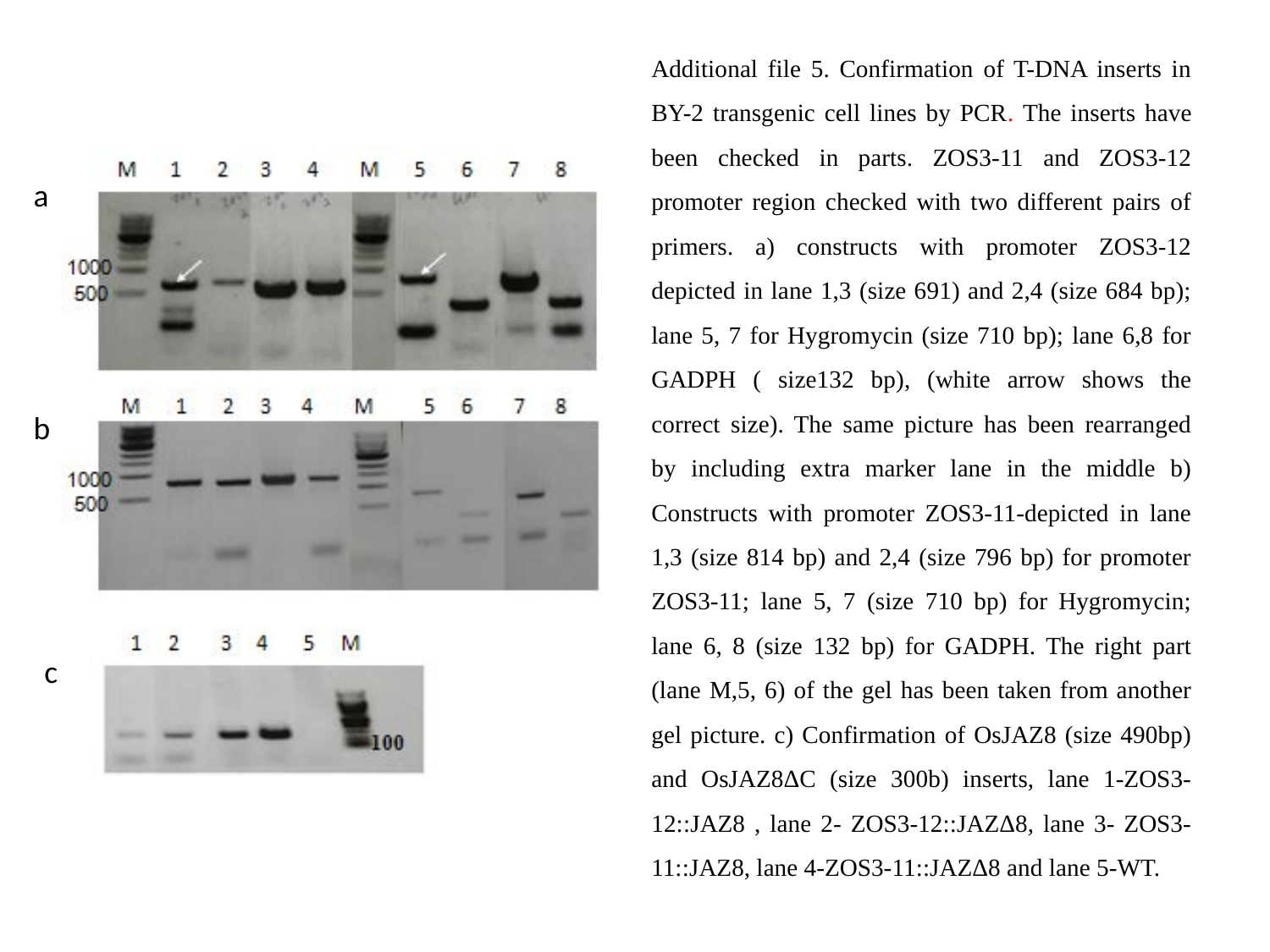

Additional file 5. Confirmation of T-DNA inserts in BY-2 transgenic cell lines by PCR. The inserts have been checked in parts. ZOS3-11 and ZOS3-12 promoter region checked with two different pairs of primers. a) constructs with promoter ZOS3-12 depicted in lane 1,3 (size 691) and 2,4 (size 684 bp); lane 5, 7 for Hygromycin (size 710 bp); lane 6,8 for GADPH ( size132 bp), (white arrow shows the correct size). The same picture has been rearranged by including extra marker lane in the middle b) Constructs with promoter ZOS3-11-depicted in lane 1,3 (size 814 bp) and 2,4 (size 796 bp) for promoter ZOS3-11; lane 5, 7 (size 710 bp) for Hygromycin; lane 6, 8 (size 132 bp) for GADPH. The right part (lane M,5, 6) of the gel has been taken from another gel picture. c) Confirmation of OsJAZ8 (size 490bp) and OsJAZ8ΔC (size 300b) inserts, lane 1-ZOS3-12::JAZ8 , lane 2- ZOS3-12::JAZΔ8, lane 3- ZOS3-11::JAZ8, lane 4-ZOS3-11::JAZΔ8 and lane 5-WT.
a
b
c

## Slide 7
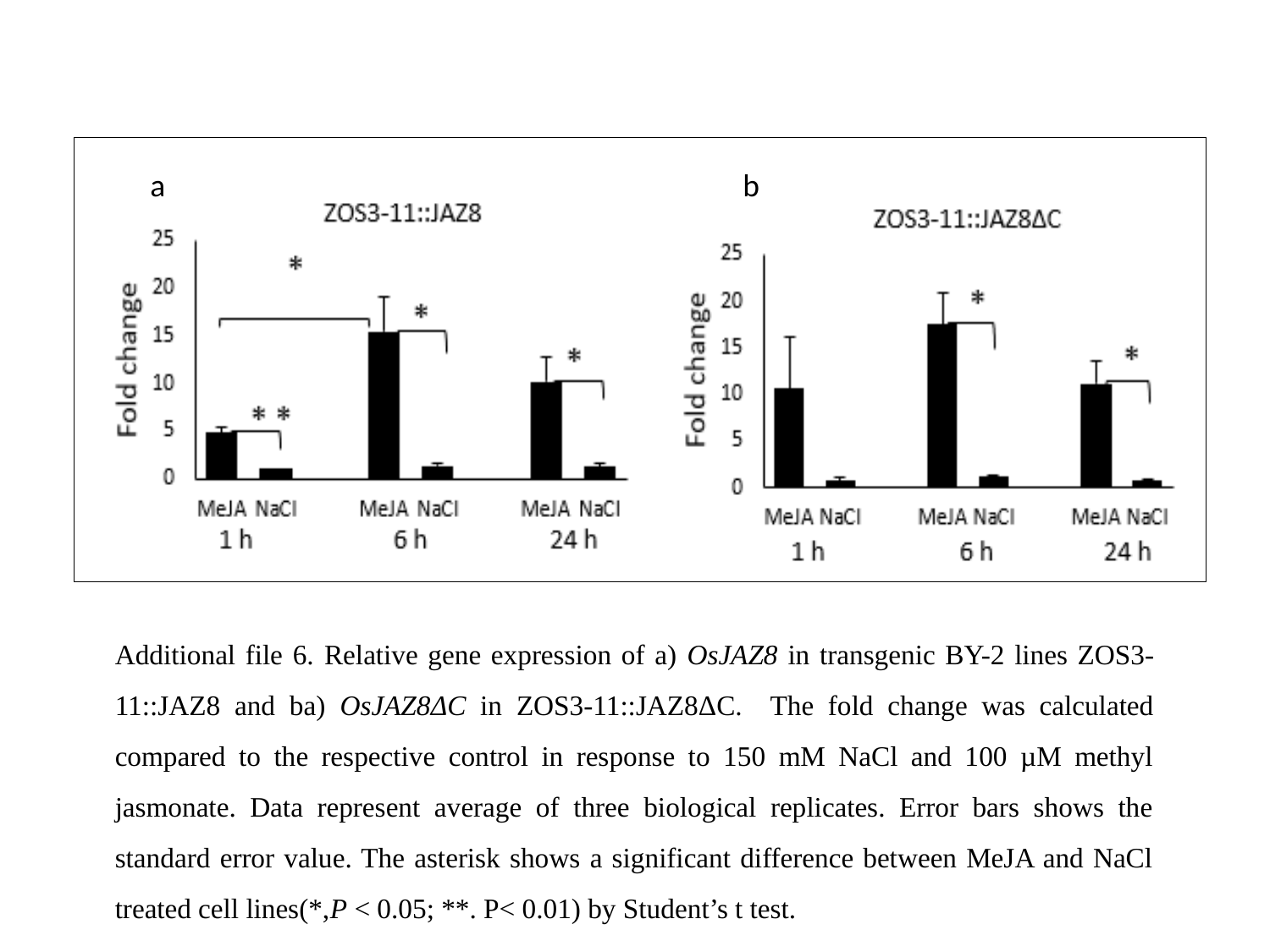

a
b
Additional file 6. Relative gene expression of a) OsJAZ8 in transgenic BY-2 lines ZOS3-11::JAZ8 and ba) OsJAZ8ΔC in ZOS3-11::JAZ8ΔC. The fold change was calculated compared to the respective control in response to 150 mM NaCl and 100 µM methyl jasmonate. Data represent average of three biological replicates. Error bars shows the standard error value. The asterisk shows a significant difference between MeJA and NaCl treated cell lines(*,P < 0.05; **. P< 0.01) by Student’s t test.

## Slide 8
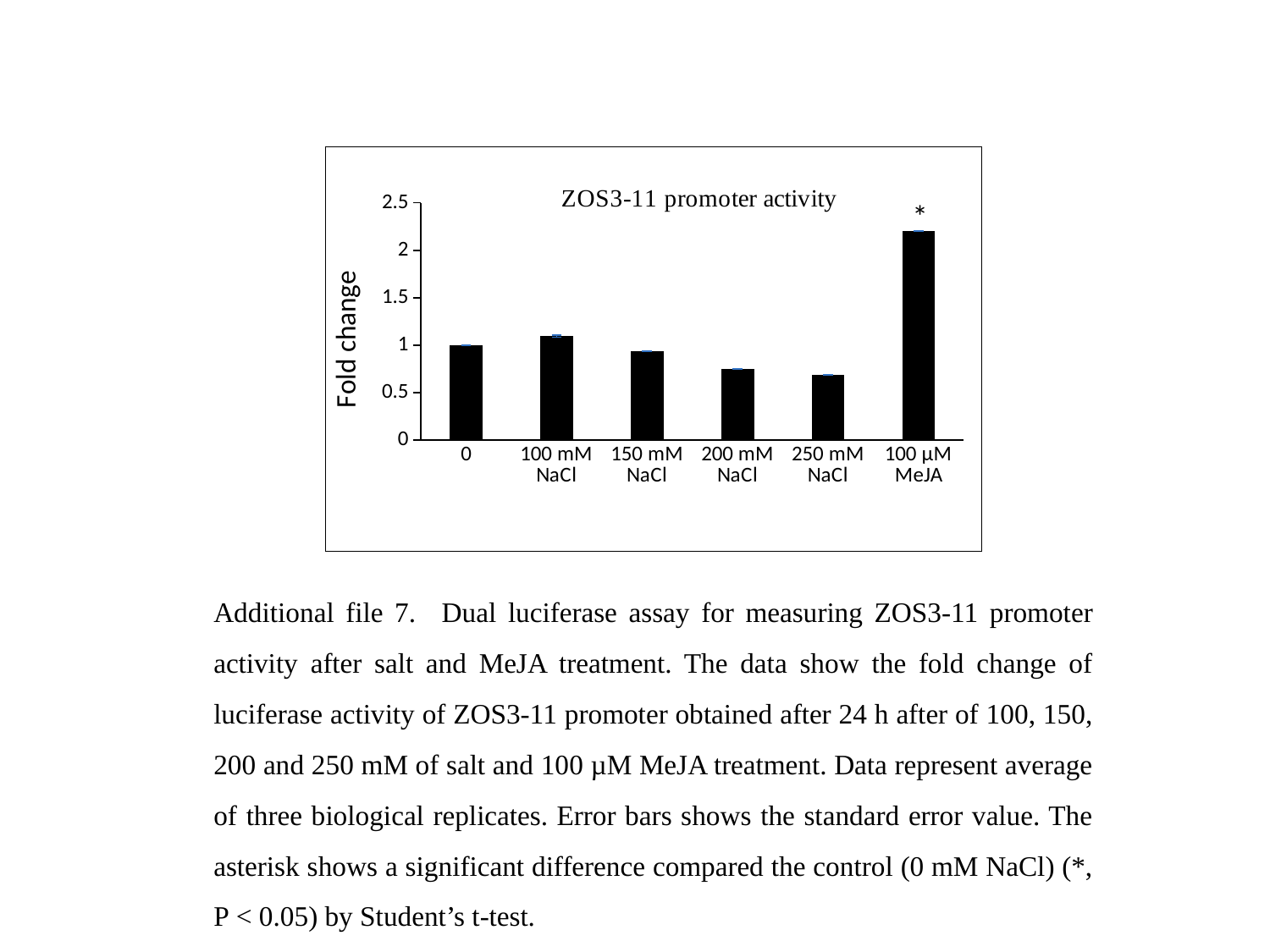

### Chart
| Category | ZOS3-11 |
|---|---|
| 0 | 1.0 |
| 100 mM NaCl | 1.09375 |
| 150 mM NaCl | 0.9333333333333333 |
| 200 mM NaCl | 0.7500000000000009 |
| 250 mM NaCl | 0.6875000000000002 |
| 100 µM MeJA | 2.2 |*
Additional file 7.   Dual luciferase assay for measuring ZOS3-11 promoter activity after salt and MeJA treatment. The data show the fold change of luciferase activity of ZOS3-11 promoter obtained after 24 h after of 100, 150, 200 and 250 mM of salt and 100 µM MeJA treatment. Data represent average of three biological replicates. Error bars shows the standard error value. The asterisk shows a significant difference compared the control (0 mM NaCl) (*, P < 0.05) by Student’s t-test.

## Slide 9
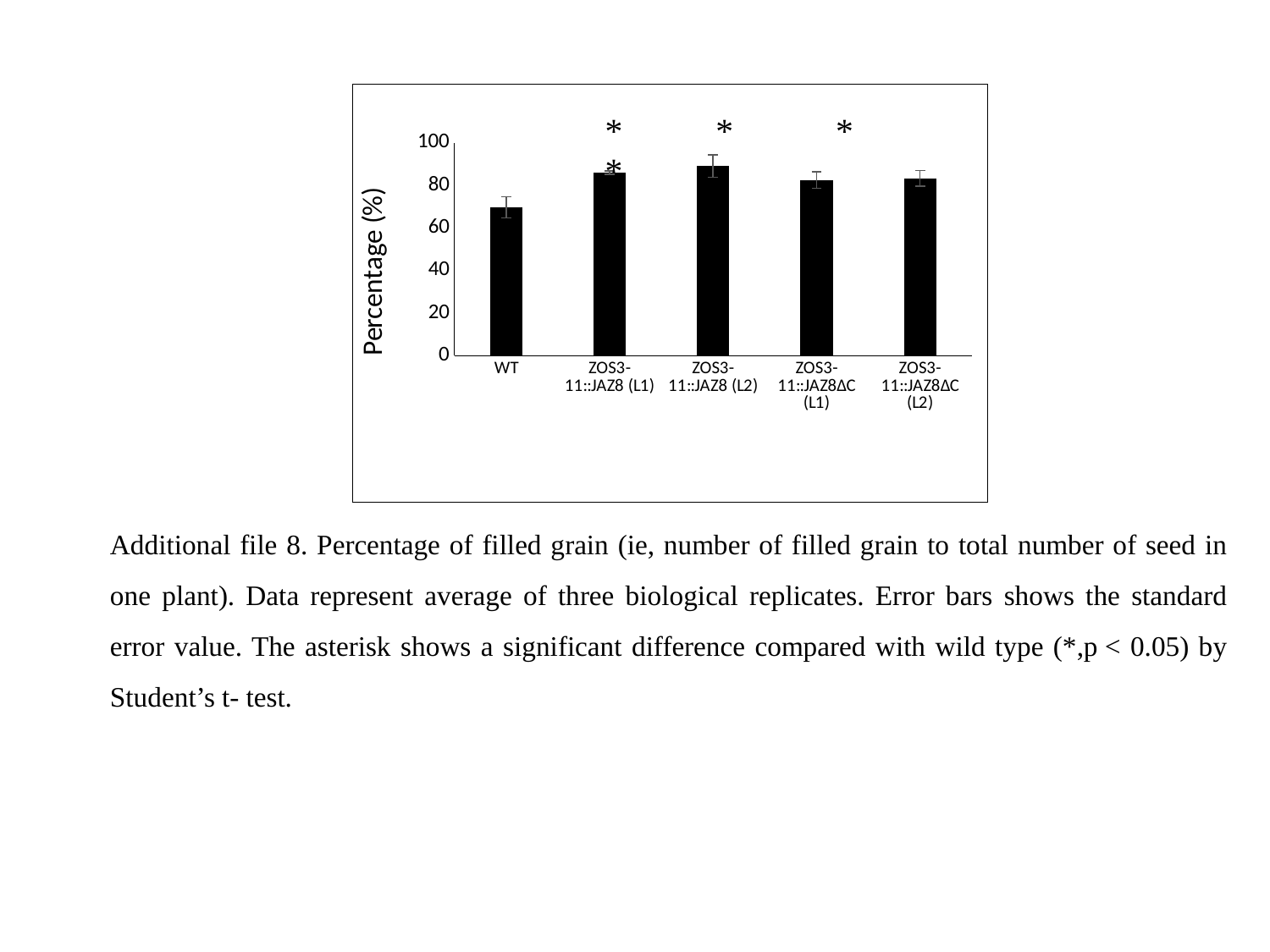

### Chart
| Category | |
|---|---|
| WT | 69.61999999999999 |
| ZOS3-11::JAZ8 (L1) | 85.99000000000002 |
| ZOS3-11::JAZ8 (L2) | 89.04 |
| ZOS3-11::JAZ8ΔC (L1) | 82.51 |
| ZOS3-11::JAZ8ΔC (L2) | 83.2 |Additional file 8. Percentage of filled grain (ie, number of filled grain to total number of seed in one plant). Data represent average of three biological replicates. Error bars shows the standard error value. The asterisk shows a significant difference compared with wild type (*,p < 0.05) by Student’s t- test.

## Slide 10
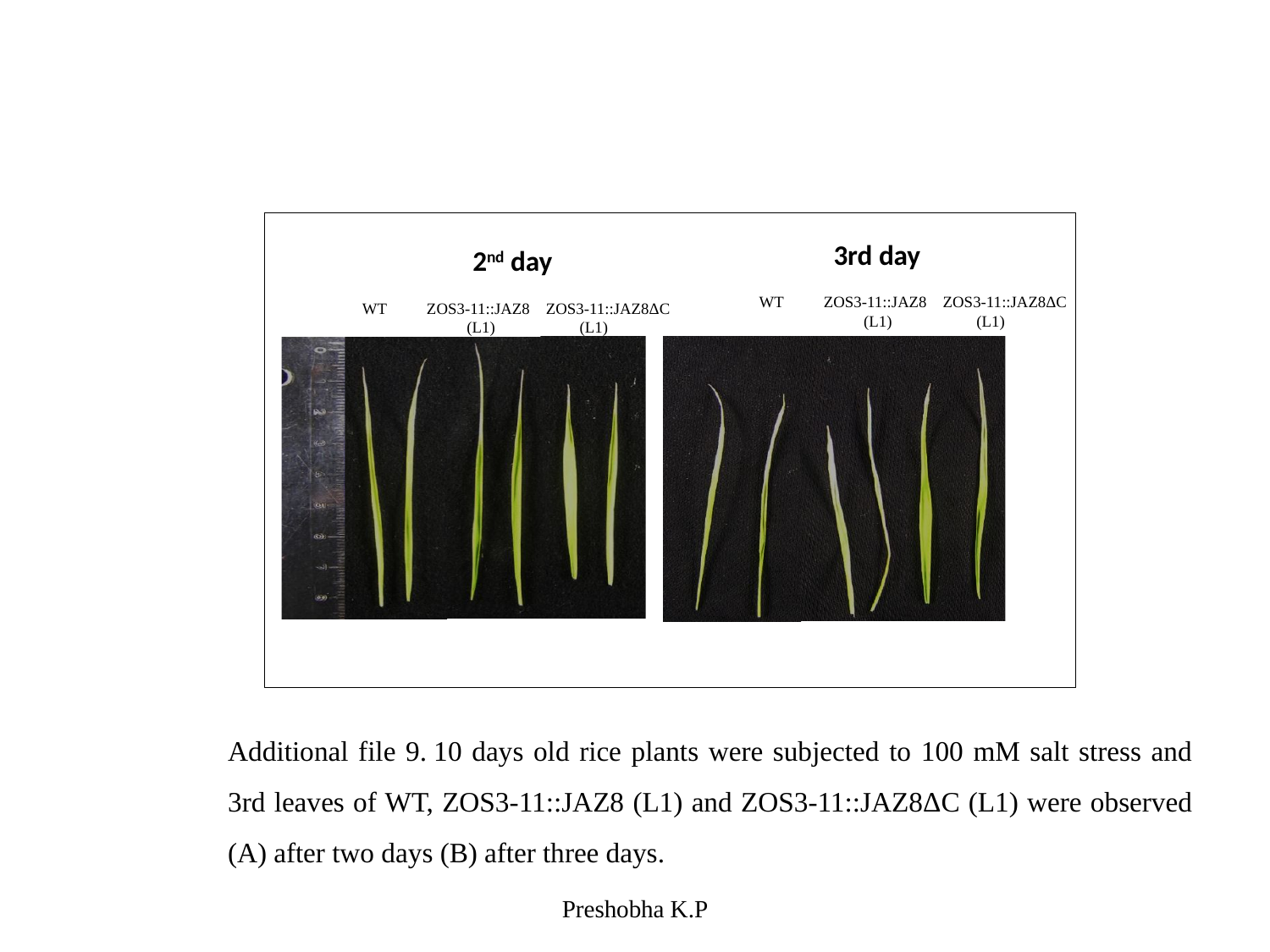

3rd day
2nd day
 WT ZOS3-11::JAZ8 ZOS3-11::JAZ8ΔC
 (L1) (L1)
 WT ZOS3-11::JAZ8 ZOS3-11::JAZ8ΔC
 (L1) (L1)
Additional file 9. 10 days old rice plants were subjected to 100 mM salt stress and 3rd leaves of WT, ZOS3-11::JAZ8 (L1) and ZOS3-11::JAZ8ΔC (L1) were observed (A) after two days (B) after three days.
Preshobha K.P

## Slide 11
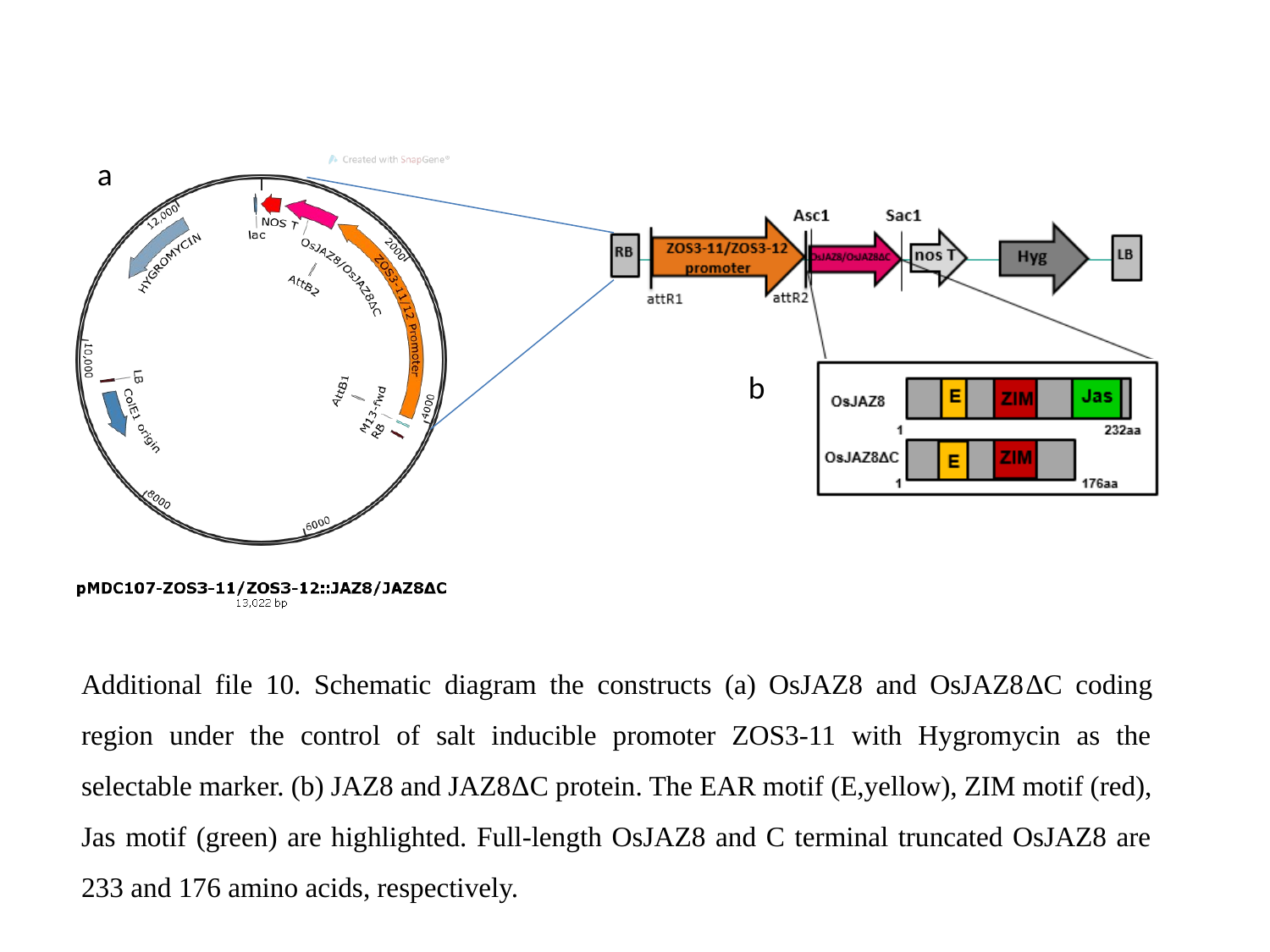

a
b
Additional file 10. Schematic diagram the constructs (a) OsJAZ8 and OsJAZ8ΔC coding region under the control of salt inducible promoter ZOS3-11 with Hygromycin as the selectable marker. (b) JAZ8 and JAZ8ΔC protein. The EAR motif (E,yellow), ZIM motif (red), Jas motif (green) are highlighted. Full-length OsJAZ8 and C terminal truncated OsJAZ8 are 233 and 176 amino acids, respectively.

## Slide 12
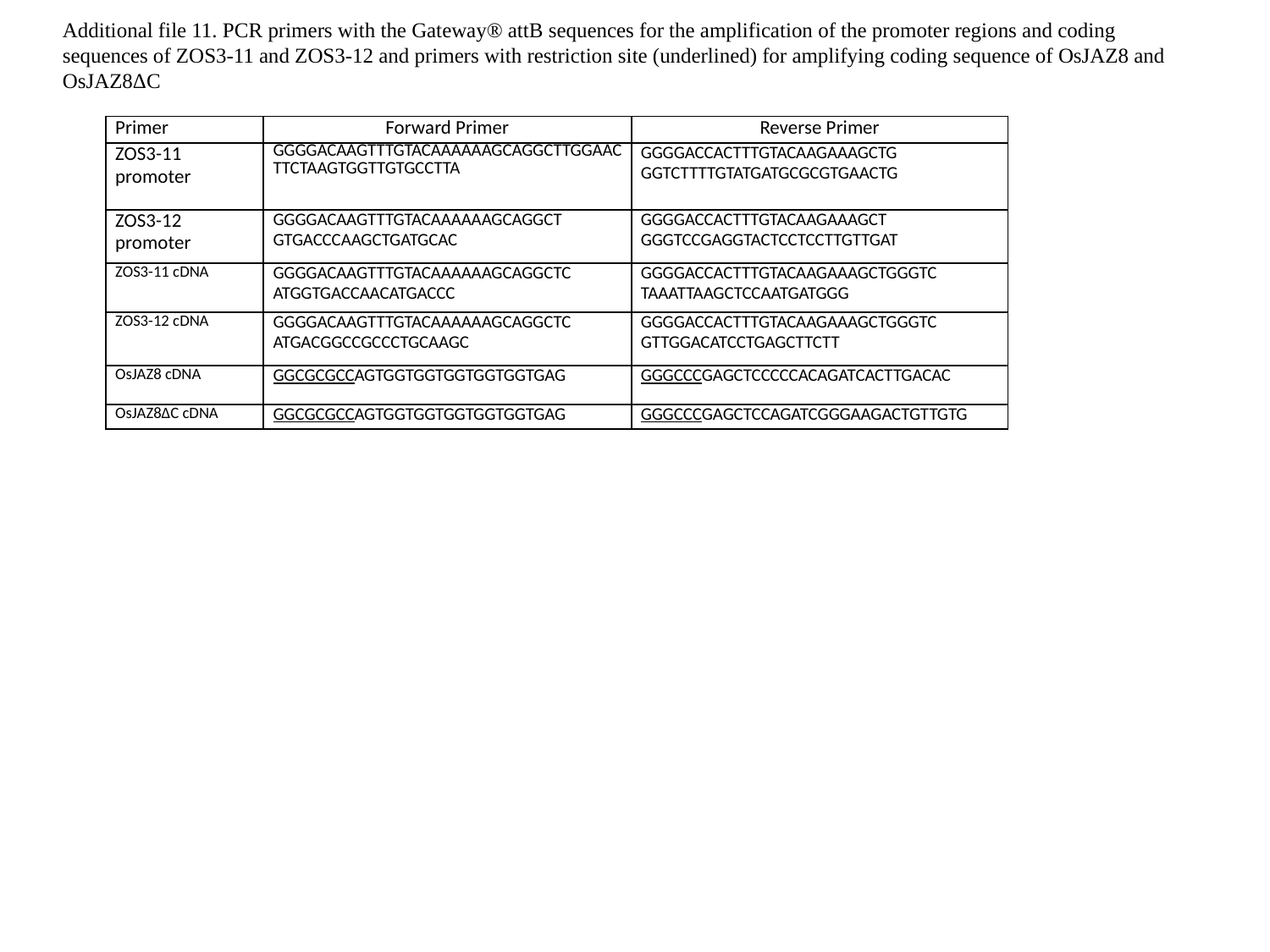

Additional file 11. PCR primers with the Gateway® attB sequences for the amplification of the promoter regions and coding sequences of ZOS3-11 and ZOS3-12 and primers with restriction site (underlined) for amplifying coding sequence of OsJAZ8 and OsJAZ8ΔC
| Primer | Forward Primer | Reverse Primer |
| --- | --- | --- |
| ZOS3-11 promoter | GGGGACAAGTTTGTACAAAAAAGCAGGCTTGGAACTTCTAAGTGGTTGTGCCTTA | GGGGACCACTTTGTACAAGAAAGCTG GGTCTTTTGTATGATGCGCGTGAACTG |
| ZOS3-12 promoter | GGGGACAAGTTTGTACAAAAAAGCAGGCT GTGACCCAAGCTGATGCAC | GGGGACCACTTTGTACAAGAAAGCT GGGTCCGAGGTACTCCTCCTTGTTGAT |
| ZOS3-11 cDNA | GGGGACAAGTTTGTACAAAAAAGCAGGCTC ATGGTGACCAACATGACCC | GGGGACCACTTTGTACAAGAAAGCTGGGTC TAAATTAAGCTCCAATGATGGG |
| ZOS3-12 cDNA | GGGGACAAGTTTGTACAAAAAAGCAGGCTC ATGACGGCCGCCCTGCAAGC | GGGGACCACTTTGTACAAGAAAGCTGGGTC GTTGGACATCCTGAGCTTCTT |
| OsJAZ8 cDNA | GGCGCGCCAGTGGTGGTGGTGGTGGTGAG | GGGCCCGAGCTCCCCCACAGATCACTTGACAC |
| OsJAZ8ΔC cDNA | GGCGCGCCAGTGGTGGTGGTGGTGGTGAG | GGGCCCGAGCTCCAGATCGGGAAGACTGTTGTG |

## Slide 13
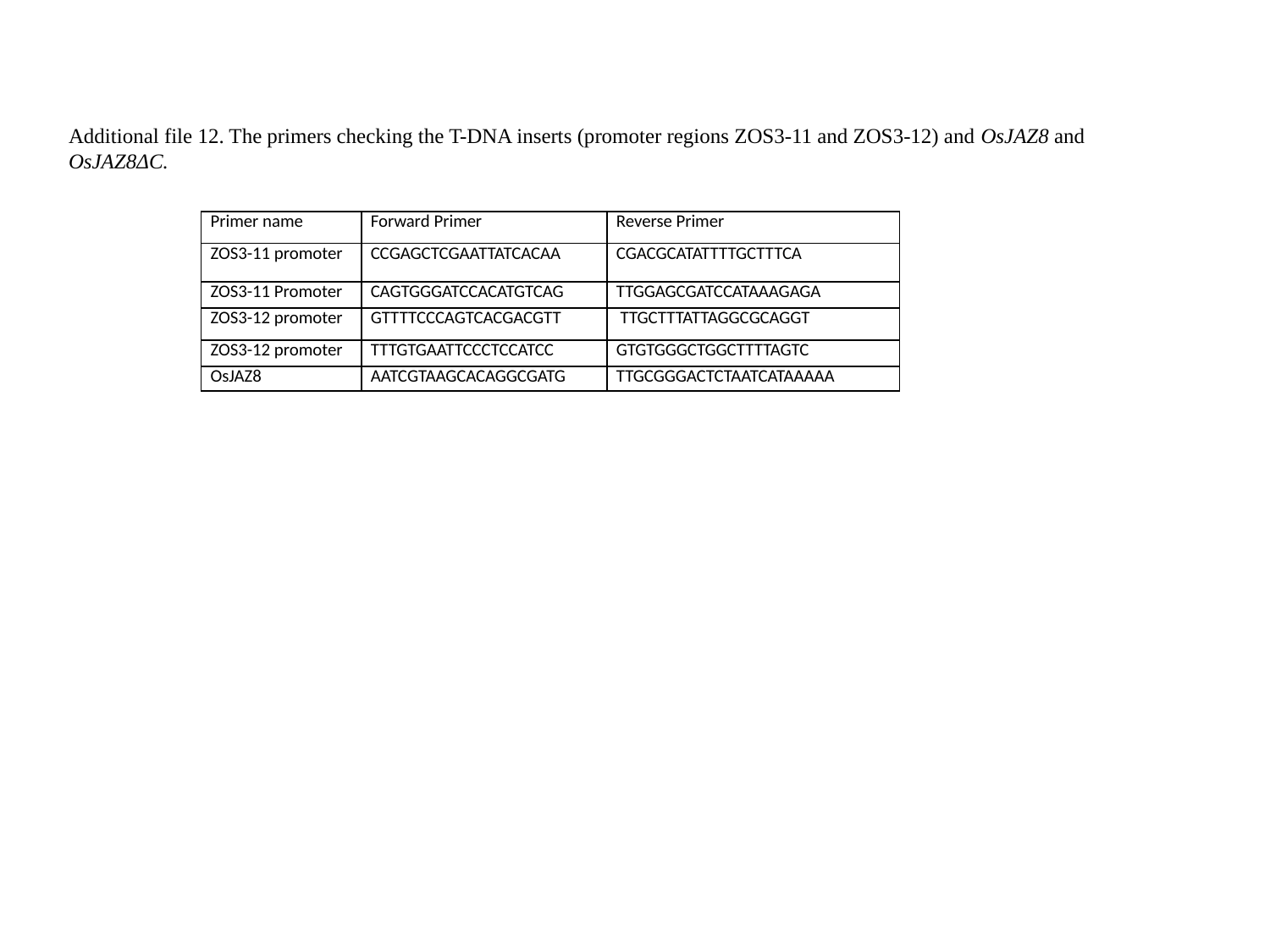

Additional file 12. The primers checking the T-DNA inserts (promoter regions ZOS3-11 and ZOS3-12) and OsJAZ8 and OsJAZ8ΔC.
| Primer name | Forward Primer | Reverse Primer |
| --- | --- | --- |
| ZOS3-11 promoter | CCGAGCTCGAATTATCACAA | CGACGCATATTTTGCTTTCA |
| ZOS3-11 Promoter | CAGTGGGATCCACATGTCAG | TTGGAGCGATCCATAAAGAGA |
| ZOS3-12 promoter | GTTTTCCCAGTCACGACGTT | TTGCTTTATTAGGCGCAGGT |
| ZOS3-12 promoter | TTTGTGAATTCCCTCCATCC | GTGTGGGCTGGCTTTTAGTC |
| OsJAZ8 | AATCGTAAGCACAGGCGATG | TTGCGGGACTCTAATCATAAAAA |

## Slide 14
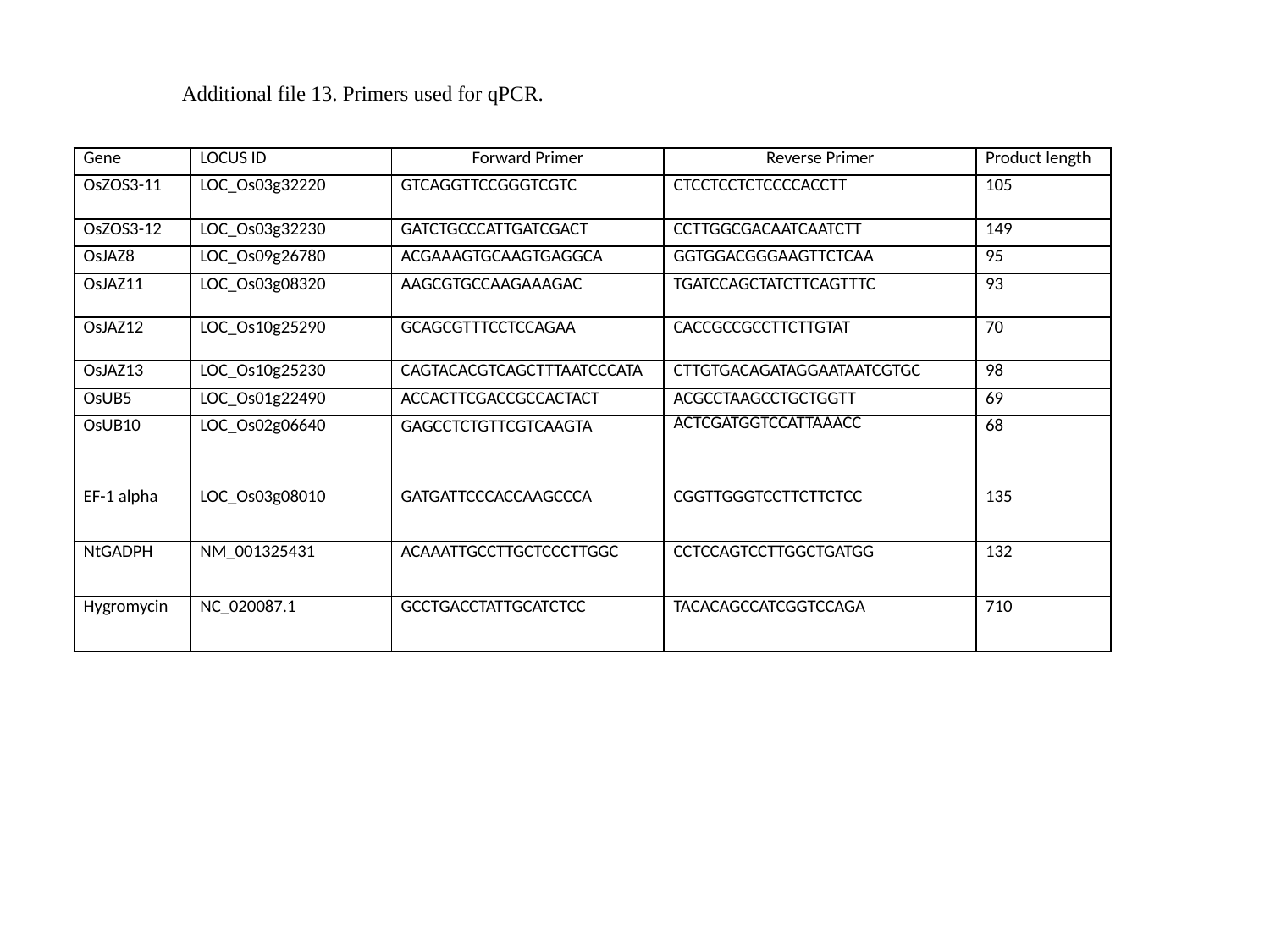

Additional file 13. Primers used for qPCR.
| Gene | LOCUS ID | Forward Primer | Reverse Primer | Product length |
| --- | --- | --- | --- | --- |
| OsZOS3-11 | LOC\_Os03g32220 | GTCAGGTTCCGGGTCGTC | CTCCTCCTCTCCCCACCTT | 105 |
| OsZOS3-12 | LOC\_Os03g32230 | GATCTGCCCATTGATCGACT | CCTTGGCGACAATCAATCTT | 149 |
| OsJAZ8 | LOC\_Os09g26780 | ACGAAAGTGCAAGTGAGGCA | GGTGGACGGGAAGTTCTCAA | 95 |
| OsJAZ11 | LOC\_Os03g08320 | AAGCGTGCCAAGAAAGAC | TGATCCAGCTATCTTCAGTTTC | 93 |
| OsJAZ12 | LOC\_Os10g25290 | GCAGCGTTTCCTCCAGAA | CACCGCCGCCTTCTTGTAT | 70 |
| OsJAZ13 | LOC\_Os10g25230 | CAGTACACGTCAGCTTTAATCCCATA | CTTGTGACAGATAGGAATAATCGTGC | 98 |
| OsUB5 | LOC\_Os01g22490 | ACCACTTCGACCGCCACTACT | ACGCCTAAGCCTGCTGGTT | 69 |
| OsUB10 | LOC\_Os02g06640 | GAGCCTCTGTTCGTCAAGTA | ACTCGATGGTCCATTAAACC | 68 |
| EF-1 alpha | LOC\_Os03g08010 | GATGATTCCCACCAAGCCCA | CGGTTGGGTCCTTCTTCTCC | 135 |
| NtGADPH | NM\_001325431 | ACAAATTGCCTTGCTCCCTTGGC | CCTCCAGTCCTTGGCTGATGG | 132 |
| Hygromycin | NC\_020087.1 | GCCTGACCTATTGCATCTCC | TACACAGCCATCGGTCCAGA | 710 |
